# Urban tree canopy fragmentation and child mortality in low- and middle-income countries

**DOI:** 10.1073/pnas.2606669123

**Published:** 2026-08-21

**Authors:** Dengkai Chi, Daniel Richards, Gabriele Manoli, Jun Yang, John S. Ji, Ye Zhang, Brenda Lin, Amy Hahs, Congqiang Liu, Yue Zhu, Yeshan Qiu, Jing Wang, Paolo Burlando, Simone Fatichi, Puay Yok Tan

**Affiliations:** ^a^ School of Earth System Science, Institute of Surface-Earth System Science, Tianjin University, Tianjin 300072, China; ^b^ Singapore–ETH Centre, Future Cities Laboratory Global, Singapore 138602, Singapore; ^c^ Bioeconomy Science Institute, Lincoln 7608, New Zealand; ^d^ Laboratory of Urban and Environmental Systems, École Polytechnique Fédérale de Lausanne, Lausanne 1015, Switzerland; ^e^ Department of Earth System Science, Institute for Global Change Studies, Ministry of Education Ecological Field Station for East Asian Migratory Birds, Tsinghua University, Beijing 100084, China; ^f^ Vanke School of Public Health, Tsinghua University, Beijing 100084, China; ^g^ School of Architecture, Tsinghua University, Beijing 100084, China; ^h^ Commonwealth Scientific and Industrial Research Organisation Environment, Dutton Park, QLD 4102, Australia; ^i^ School of Agriculture, Food and Ecosystem Sciences, Burnley Campus, The University of Melbourne, Melbourne, VIC 3121, Australia; ^j^ State Key Laboratory of Internet of Things for Smart City, Department of Arts and Design, University of Macau, Macao Special Administrative Region 999078, China; ^k^Department of Architecture, College of Design and Engineering, National University of Singapore, Singapore 117566, Singapore; ^l^ Institute of Environmental Engineering, Eidgenössische Technische Hochschule Zurich, Zurich 8093, Switzerland; ^m^Department of Civil and Environmental Engineering, College of Design and Engineering, National University of Singapore, Singapore 117576, Singapore

**Keywords:** aggregation, counterfactual scenario analysis, DHS, green space configuration, under-5 mortality

## Abstract

Exposure to urban green spaces is associated with lower risks of several leading causes of under-5 mortality (U5M). While urban development improves infrastructure, healthcare access, and socioeconomic conditions, rapid urbanization in low- and middle-income countries (LMICs) has also accelerated the loss and fragmentation of green spaces. Such fragmentation may weaken ecosystem services critical for child health and increase exposure to vector-borne infections, yet its implications for U5M remain poorly understood and must be weighed against the benefits of urbanization. Here, we quantified associations between urban tree canopy cover and canopy aggregation with all-cause U5M using individual-level longitudinal mortality data between 2000 and 2019 from 57 LMICs, and identified priority areas for expanding canopy cover and improving canopy aggregation across 98 LMICs using counterfactual scenario analyses. The association between canopy cover and U5M was nonlinear, with risk declining steeply at low-to-intermediate levels of canopy cover. At a given level of canopy cover, every 1% increase in tree-to-tree adjacency was associated with a 0.17% reduction in mortality risk (hazard ratio: 0.9983, 95% CI: 0.9972 to 0.9995). The largest contiguous high-priority areas were concentrated in the West Sudanian Savanna, the eastern Punjab region of Pakistan, and the Ganges–Brahmaputra Delta in Bangladesh, where high U5M burdens align with substantial potential to improve tree canopy. This study establishes a robust association between tree canopy characteristics and U5M across LMICs. We identify canopy cover and fragmentation as potentially modifiable risk factors, highlighting important implications of urban greening design for delivering cobenefits for child health and climate mitigation.

## Significance

Although international health agendas increasingly promote nature-based solutions, little is known about whether the spatial configuration of urban trees associates with child survival. Using longitudinal data from more than 420,000 children across 57 LMICs, we show that both greater tree cover and lower canopy fragmentation are independently associated with reduced U5M. Counterfactual scenario analyses highlight the West Sudanian Savanna, the eastern Punjab region of Pakistan, and the Ganges–Brahmaputra Delta in Bangladesh as high-priority regions where improving tree canopy conditions may yield the greatest potential for alleviating the U5M burden. Our findings identify tree canopy fragmentation as a potentially modifiable environmental risk factor for child survival and highlight urban greening as a scalable strategy with potential to reduce U5M.

Child survival has long served as an important proxy measure for overall population health and socioeconomic development (1). Despite substantial progress over recent decades, an estimated 4.8 million under-5 children died in 2023 (2) alone. The burden of these deaths is borne disproportionately by low- and middle-income countries (LMICs), particularly in Sub-Saharan Africa and Southern Asia (1). While inadequate access to essential, life-saving interventions remains the direct driver of these deaths, early-life health is increasingly shaped by climate stress (3), air pollution (4, 5), and loss of green spaces (6–9), which alter the biophysical and social contexts in which children develop. Recent reports from COP29 further underscore that nature and biodiversity form the foundation of healthy societies and sustainable food systems, highlighting their critical role in safeguarding child health (10).

Green spaces, defined as areas dominated by vegetation including trees, shrubs, and grasses, can influence child health through multiple pathways driven by ecosystem services (11, 12). First, green spaces mitigate environmental hazards by reducing heat, air pollution, and noise exposure, thereby improving ambient environmental quality (13, 14). Second, contact with green spaces can alleviate psychological stress, restore attention capacity, and promote maternal mental well-being (15). Third, green spaces provide opportunities for physical activity and social cohesion, which are important determinants of child and maternal health (16, 17). Finally, green spaces support microbial ecosystems and microbial diversity (18), offering an additional pathway through which vegetation may improve child health. Consistent with these mechanisms, a growing body of epidemiological evidence shows that greater exposure to green spaces is associated with lower risks of preterm birth (19, 20), low birth weight (20, 21), infectious disease (6, 9, 22), and congenital anomalies (23, 24), which are the leading causes of under-5 mortality (U5M) worldwide (1).

Most of the existing evidence comes from high-income regions (25, 26) and only one study has investigated the link between green space exposure and U5M in an upper middle-income country (8). Moreover, our understanding of green space impacts on under-5 health is focused on measures of green space quantity, such as remote sensing derived greenness indices (e.g., normalized difference vegetation index), the proportion of green land cover or tree density. The literature therefore largely overlooks the role that the spatial configuration of green spaces can play in influencing U5M (27). This represents a crucial policy gap, because rapid urbanization in many LMICs has led not only to the loss of natural environments but also to their increasing fragmentation (28, 29). Fragmented green spaces have reduced ecological functioning and capacity to mitigate environmental hazards such as extreme heat (30) and air pollution (31). Fragmentation also disrupts habitats and ecological corridors that facilitate species movement, thereby undermining biodiversity (32), which underpins many ecosystem services vital to human health (33, 34). Moreover, habitat fragmentation can intensify edge effects and increase spatial overlap between human settlements and vector or pathogen reservoirs, potentially elevating exposure to infectious diseases (35, 36). In addition, less aggregated and poorly connected green spaces, lacking linkages provided by street trees and linear green corridors, may reduce both the frequency and duration of residents’ use of urban green areas, thereby diminishing social cohesion and opportunities for physical activity such as walking, biking, and jogging (37).

Recent research linking green space fragmentation to health outcomes in high-income cities show that the fragmentation of green spaces, and not just their quantity, associates with mortality risk (38–40), mental health (40), and noncommunicable diseases (41) in adults. For example, a nationwide Swiss longitudinal study revealed that more aggregated canopies were associated with lower risks of natural-cause and multiple cause-specific mortality in adults, independent of total cover (42). Yet, evidence for children whose exposure windows, mobility, and physiological vulnerability differ profoundly from adults is virtually absent, particularly longitudinal evidence (27). To date, only one cross-sectional study for Georgia, United States, has demonstrated that higher green space aggregation was linked to lower risk of one cause of U5M (i.e., preterm birth) at the census tract level (7). If the spatial configuration of urban green space can be designed to benefit under-5 health outcomes, substantial health gains could be achieved for the same area of urban green space created. There is therefore a need to provide robust evidence about the associations of green spaces aggregation with U5M to inform more effective greening strategies and accelerate progress toward the Sustainable Development Goal of ending preventable child deaths in LMICs.

In recent years, many LMICs have launched ambitious large-scale tree planting programs, such as Pakistan’s Ten Billion Tree Tsunami Programme, Ethiopia’s Green Legacy Initiative, and India’s Haritha Haram. These programs are primarily aimed at addressing land degradation and climate change, with health benefits typically framed as ancillary cobenefits rather than explicit programme targets (43). A recent review calls for more explicit integration of health goals into nature-based solutions by placing community well-being at the center of conservation and restoration efforts in LMICs, and highlights the need to evaluate a broader range of health-relevant outcomes to fully capture the benefits of these interventions (44). However, quantitative evidence remains limited on how improvements in canopy cover and fragmentation could translate into child health benefits, where such improvements may yield the greatest potential health gains, and how these gains can be systematically evaluated under counterfactual greening scenarios. Such evidence is particularly important for resource-limited LMICs, where land, funding, and institutional capacity for large-scale greening strategies are often constrained.

Here, we evaluate the associations between both the quantity and spatial aggregation of tree canopy cover and U5M in LMICs. By linking fine-scale annual tree canopy cover data with longitudinal Demographic and Health Survey (DHS) data (45) (~420,000 children across 57 LMICs, 2000–2019), we first use time-varying Cox regression models to quantify the exposure–response relationships between canopy cover, canopy aggregation, and U5M. We then integrate these exposure–response functions into a counterfactual scenario framework to identify priority areas for increasing canopy cover and reducing fragmentation across 98 LMICs, based on estimated health gains under alternative greening scenarios. Together, these analyses provide robust longitudinal, cross-country evidence on how tree canopy fragmentation, beyond the total cover, associates with child survival in LMICs and introduce a transferable framework for evaluating how the spatial arrangement of trees may support child health, offering actionable insights for prioritizing greening strategies to maximize health benefits in resource-constrained settings.

## Results

### U5M and Tree Canopy Characteristics in LMICs.

There were 57 LMICs included in our analysis; 34 in Sub-Saharan Africa, seven in Latin America and the Caribbean, five in the East Asia and Pacific region, five in the Europe and Central Asia, three in Middle East and North Africa, and three in South Asia (*SI Appendix*, *Materials and Methods* and *Mortality Data* and *Screening of DHS Data* and Table S1). Among these countries, 133 surveys were conducted across 43,373 urban clusters between 2000 and 2019. A total of 420,244 children were included in the analysis, with 11,641,162 person-months and 16,988 all-cause deaths. Among the deaths, 85.2% occurred within 12 mo after birth.

**Fig. 1.**
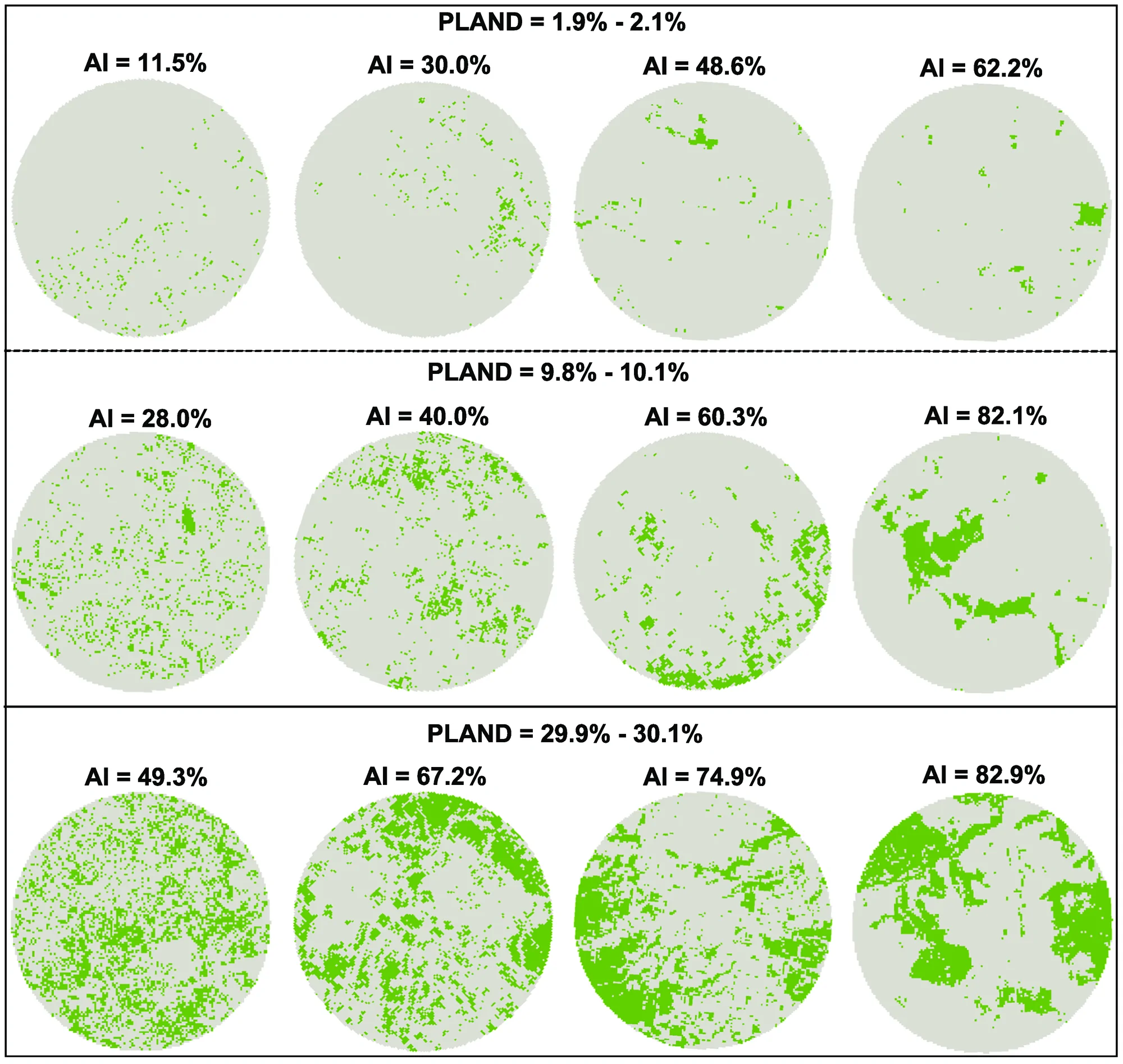
Illustrative urban clusters (~2 km buffers) in the Demographic and Health Surveys (DHS) demonstrating differences in tree canopy coverage (PLAND) and spatial aggregation (AI). Tree-covered areas are highlighted in green.

We calculated tree canopy coverage (PLAND, the percentage of tree canopy cover) and the aggregation index (AI) within a 2 km radius for each cluster for each of the 5 y preceding the survey (*SI Appendix*, Table S2). These measures are commonly used in landscape ecological studies to investigate the relationships between landscape patterns, processes, and functions (29, 46). The AI measures how physically connected or clumped together patches of the same land cover type are across a landscape, by measuring the proportion of all possible adjacencies between pixels of the same class that actually occur (46) (*SI Appendix*, *Materials and Methods and Tree Canopy Characteristics* and Fig. S1). High AI values indicate that tree canopy patches form large, continuous, well-connected areas, while low AI values indicate that the canopy is fragmented into many small, isolated patches (Fig. 1). The mean (±SD) of the tree canopy metrics experienced by the study participants were 19.9% (±27.3%) for PLAND and 51.1% (±29.4%) for AI (*SI Appendix*, Table S3). PLAND and AI were highly correlated (*r* = 0.78), as higher tree canopy cover inevitably leads to greater canopy aggregation. Consequently, both metrics exhibited similar patterns of geographic heterogeneity, with the highest PLAND and AI values concentrated in coastal West Africa, the Congo Basin, Central America, and the Western Ghats of India, while the lowest values predominated in northern Africa and much of India (*SI Appendix*, Fig. S2).

### Associations Between Tree Canopy Characteristics and U5M.

**Fig. 2.**
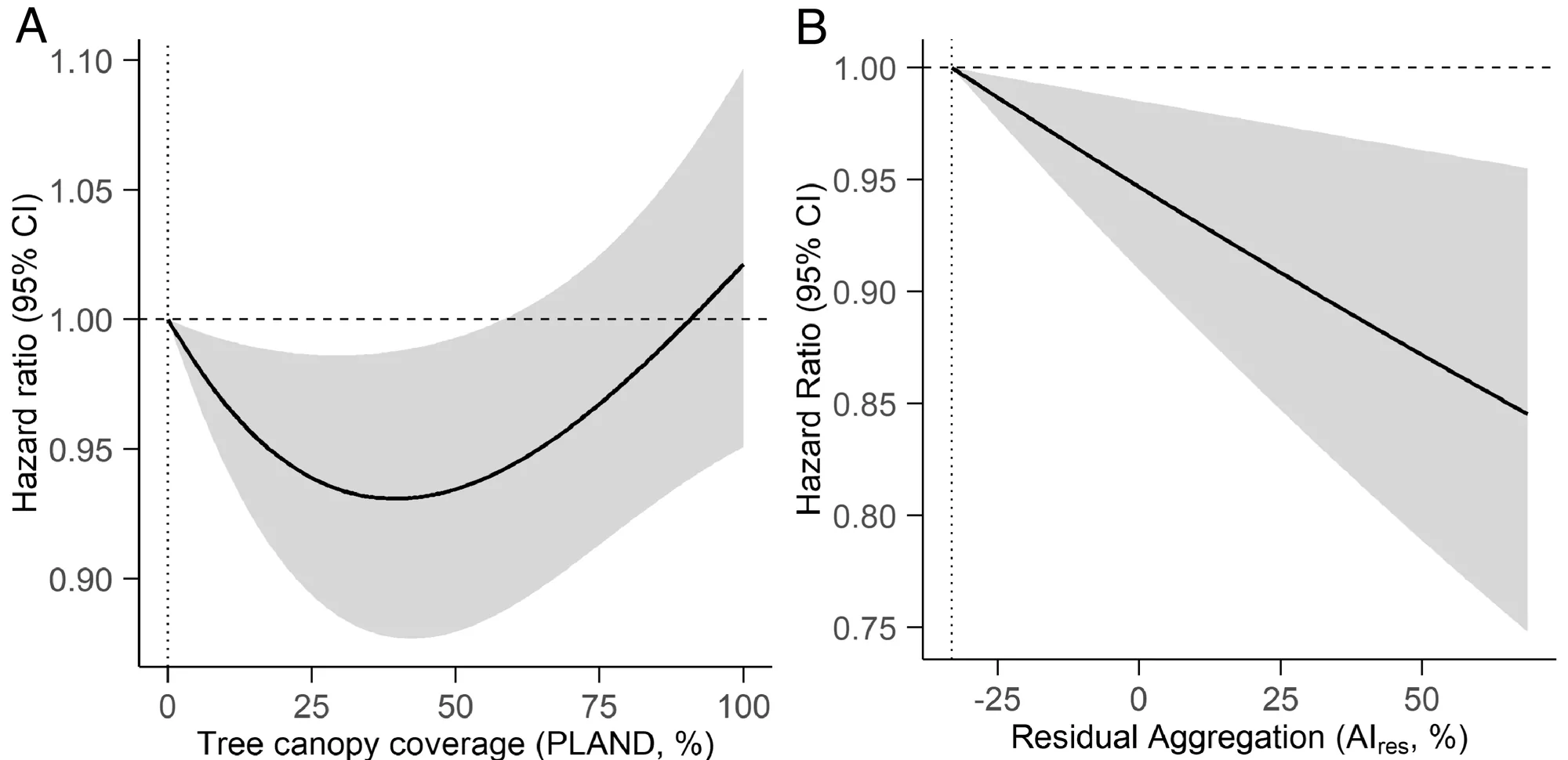
Exposure–response relationships between urban tree canopy and U5M. Exposure–response curves for (*A*) PLAND and (*B*) residual canopy aggregation (AI_res_) in relation to U5M. Associations were estimated among all eligible children under 5 y of age (*n* = 420,244) using time-varying Cox regression models. Models were adjusted for household living conditions (drinking water source, toilet facility, and cooking fuel type), household wealth, maternal characteristics (age, education, and marital status), fertility- and birth-related factors (caesarean section, place of delivery, birth order, breastfeeding, and twin status), and environmental conditions (climate region and annual mean PM_2.5_ concentration). Child sex and survey were included as strata. Effect estimates are hazard ratios with 95% CI, with robust SE clustered at the individual child level.

To quantify the independent associations of PLAND and AI with U5M, we jointly modeled PLAND and residual aggregation (AI_res_) in time-varying Cox regression models, adjusting for household living conditions, maternal characteristics, and fertility- and birth-related factors (*SI Appendix*, *Materials and Methods* and *Statistical Analyses*). AI_res_ was defined as the residuals from regressing AI on PLAND, representing the component of canopy aggregation independent of canopy amount (*SI Appendix*, *Materials and Methods* and *Statistical Analyses*). For PLAND, the association shows a nonlinear pattern (Fig. 2*A*). Mortality risk declined sharply as PLAND increased from 0 % to around 30%, beyond which the association flattened and then rose at very high coverage levels. The lowest estimated hazard ratio (HR) occurred at 39.7% canopy coverage, corresponding to a 6.9% reduction in HR compared with areas without trees. In contrast, AI_res_ showed a linear association with U5M (Fig. 2*B*). Each percentage point increase in AI_res_, representing one-percentage-point increase in canopy aggregation relative to the level expected given the same canopy cover, was associated with a 0.17% reduction in mortality risk (HR: 0.9983, 95% CI: 0.9972 to 0.9995) (see *SI Appendix*, Table S4 for full model estimates).

The associations were robust across a series of sensitivity analyses (*SI Appendix*, *Materials and Methods* and *Statistical Analyses*). When we evaluated two alternative configuration metrics related to fragmentation used in the landscape ecology literature, the aggregation metric PLADJ (the percentage of like adjacencies) also showed a protective association with a same effect size as AI (*SI Appendix*, Fig. S3*A*), whereas the fragmentation metric PD (patch density) was positively associated with U5M (HR: 1.0045, 95% CI: 1.0015 to 1.0075) (*SI Appendix*, Fig. S3*B*), consistent with our main interpretation for AI. The magnitude and shape of the exposure–response curves for both PLAND and AI_res_ also remained stable after adjustment for additional covariates, including annual mean air temperature (*SI Appendix*, Fig. S4), the coverage of other vegetation types (e.g., shrub, grass; *SI Appendix*, Fig. S5 and Table S5), motorized travel time to healthcare (*SI Appendix*, Fig. S6), and sampling weights (*SI Appendix*, *Materials and Methods* and *Statistical Analyses* and Fig. S7). Leave-one-country-out analyses did not identify any single country that materially altered the overall exposure–response relationship (*SI Appendix*, Fig. S8 and Table S6), indicating that the findings were not driven by any individual country. In addition, incorporating prenatal exposure through lagged analyses attenuated the estimated associations, while the overall exposure–response patterns remained broadly similar to those observed in the primary analyses (*SI Appendix*, *Materials and Methods* and *Statistical Analyses* and Fig. S9).

### Modification Effects of Individual and Cluster-Level Factors.

**Fig. 3.**
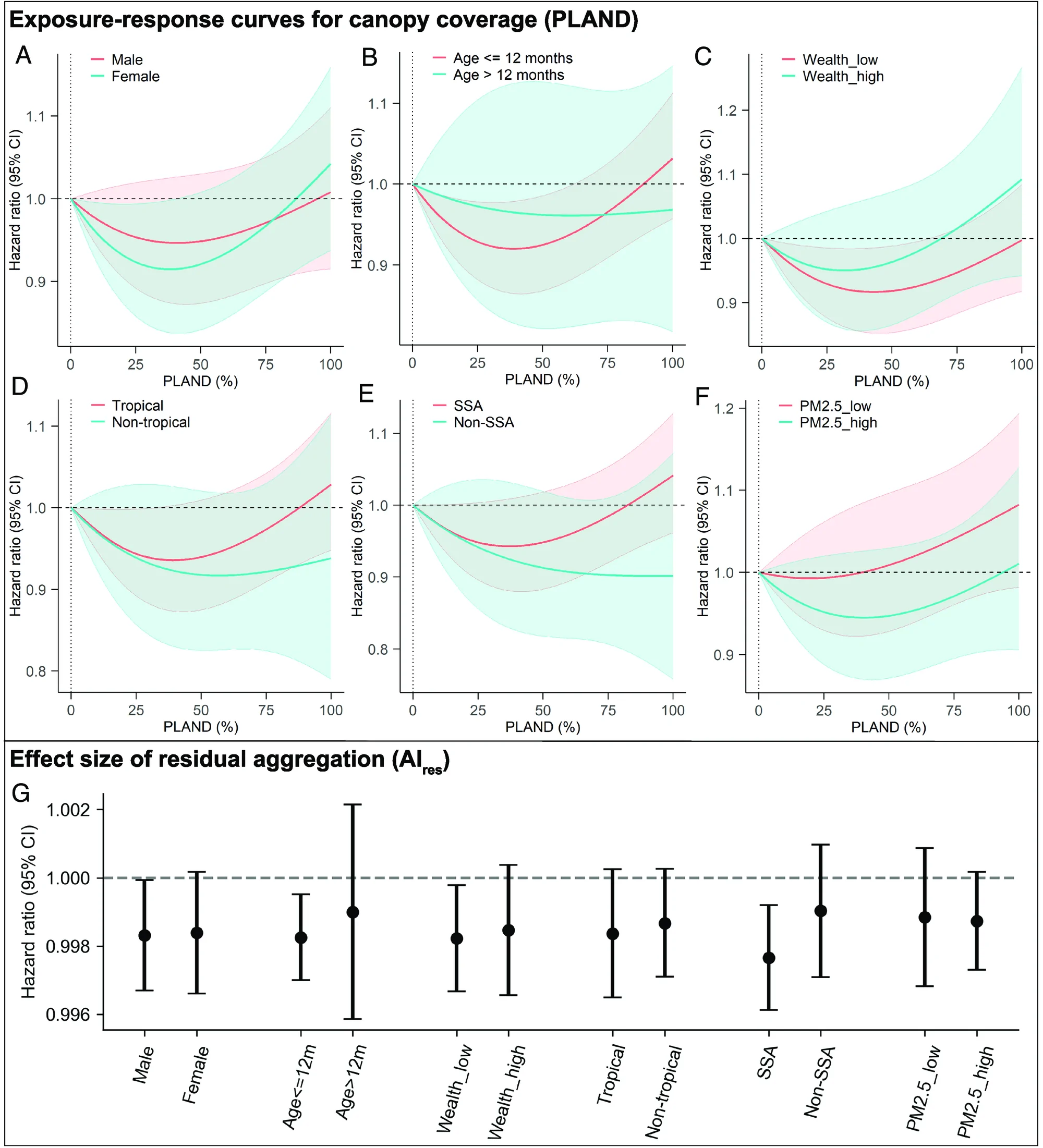
Exposure–response relationships between urban tree canopy and U5M in subgroups. Exposure–response curves for (*A*–*F*) tree canopy coverage (PLAND) and (*G*) residual canopy aggregation (AI_res_) in relation to U5M. Associations were estimated among eligible children under 5 y of age in the subgroups divided by child sex (male, *n* = 215,853 vs. female, *n* = 204,391), age group (≤12 mo, *n* = 404,673 vs. >12 mo, *n* = 311,930), household wealth (lowest three, *n* = 217,486 vs. highest three quintiles *n* = 202, 758, based on the regenerated six-quintile wealth index), climate zones (tropical, *n* = 217,885 vs. nontropical, *n* = 202,359), geographical region (SSA, *n* = 208,915 vs. non-SSA, *n* = 211,329), and annual mean PM_2.5_ concentration (above, *n* = 210,231 vs. below the median of 31.5 µg/m^3^, *n* = 210,013) using time-varying Cox regression models. Models were adjusted for household living conditions (drinking water source, toilet facility, and cooking fuel type), household wealth, maternal characteristics (age, education, and marital status), fertility- and birth-related factors (caesarean section, place of delivery, birth order, breastfeeding, and twin status), and environmental conditions (climate region and annual mean PM_2.5_ concentration). Child sex and survey were included as strata. Effect estimates are hazard ratios with 95% CI, with robust SE clustered at the individual child level. SSA: Sub-Saharan Africa.

We conducted stratified analyses to assess potential effect modification by individual- and cluster-level characteristics. Higher PLAND was associated with lower mortality risk in both boys and girls, with a slightly stronger association among girls at moderate canopy cover (Fig. 3*A*), whereas AI_res_ showed comparable associations across sexes (Fig. 3*G*). Clear protective associations for both metrics were observed among infants (≤12 mo), whereas associations were weaker and nonsignificant among children older than 12 mo (Fig. 3 *B* and *G*), likely reflecting limited statistical power given the substantially smaller number of deaths after infancy (*n* = 2,512; *SI Appendix*, Table S3). Across the household wealth groups, children from lower-wealth households tended to experience greater benefits at higher canopy cover and aggregation (lowest three wealth quintiles; see *SI Appendix*, *Materials and Methods* and *Statistical Analyses*) (Fig. 3 *C* and *G*). PLAND exhibited broadly similar exposure–response relationships across climate zones and regions at lower canopy cover, whereas stronger protective associations emerged in nontropical and non-SSA regions where canopy cover is relatively lower (Fig. 3 *D* and *E*); for AI_res_, inverse associations were most evident in SSA (Fig. 3*G*). Finally, PLAND appeared to confer greater protection in areas with higher PM_2.5_ concentrations (above the median of 31.5 µg/m^3^), whereas AI_res_ showed similar associations across PM_2.5_ strata (Fig. 3 *F* and *G*).

### Estimated Health Gains Under Counterfactual Tree Canopy Scenarios.

We estimated the potential health gains associated with improved tree canopy conditions using spatially matched estimates of U5M, PLAND, and AI within 184,685 urban and peri-urban grids (~4 × 4 km) across 55 countries included in the association analysis and another 43 LMICs (*SI Appendix*, Table S10), using 2015 as the baseline year and applying the estimated associations of PLAND and AI_res_ with U5M from the main model (Fig. 2 and *SI Appendix*, Fig. S10). We evaluated two counterfactual scenarios: first, PLAND was increased to its maximum feasible level (including existing vegetation and bare areas; *SI Appendix*, Table S5), with an upper bound of 30% motivated by the observed exposure–response relationship and the 3-30-300 tree exposure guideline, where 30 denotes 30% neighborhood tree canopy cover (47); second, we assessed additional gains associated with optimizing AI to its empirically observed upper bound (97.5th percentile) conditional on this canopy cover (*SI Appendix*, *Materials and Methods* and *Counterfactual Scenario Analysis* and Fig. S11). Baseline U5M probabilities (48) in the studied areas showed pronounced spatial heterogeneity across the countries, ranging from 0.006 to 0.130 in 2015 (*SI Appendix*, Fig. S12*A*). The highest national average was observed in the Central African Republic and the lowest in Cuba. Regionally, the largest mortality burdens were concentrated in Sub-Sahara Africa, with additional hotspots in South Asia and the East Asia and Pacific region.

We estimated that 63.5% of urban and peri-urban grids had the potential to expand tree canopy cover given existing land availability, resulting in an average increase of 9.6 percentage points and a total cover of 146,870 km^2^ (*SI Appendix*, Fig. S13*A*). Under this scenario, the estimated reduction in U5M corresponded to approximately 23,800 (95% UI: 5,069 to 44,469) fewer under-5 deaths, accounting for 1.7% of all U5M in 2015. Further, optimizing tree canopy aggregation was feasible in 60.1% of grids, with an average improvement of 32.8% in AI (*SI Appendix*, Fig. S13*B*). This level of improvement in tree-to-tree connectivity is associated with an additional reduction of approximately 53,570 (95% UI: 19,778 to 88,473) deaths (3.7% of all U5M), leading to a total of 77,369 (95% UI: 24,846 to 132,941) potential reductions in U5M, or 5.4% of all U5M in 2015 (see *SI Appendix*, Fig. S14 for the spatial distribution).

**Fig. 4.**
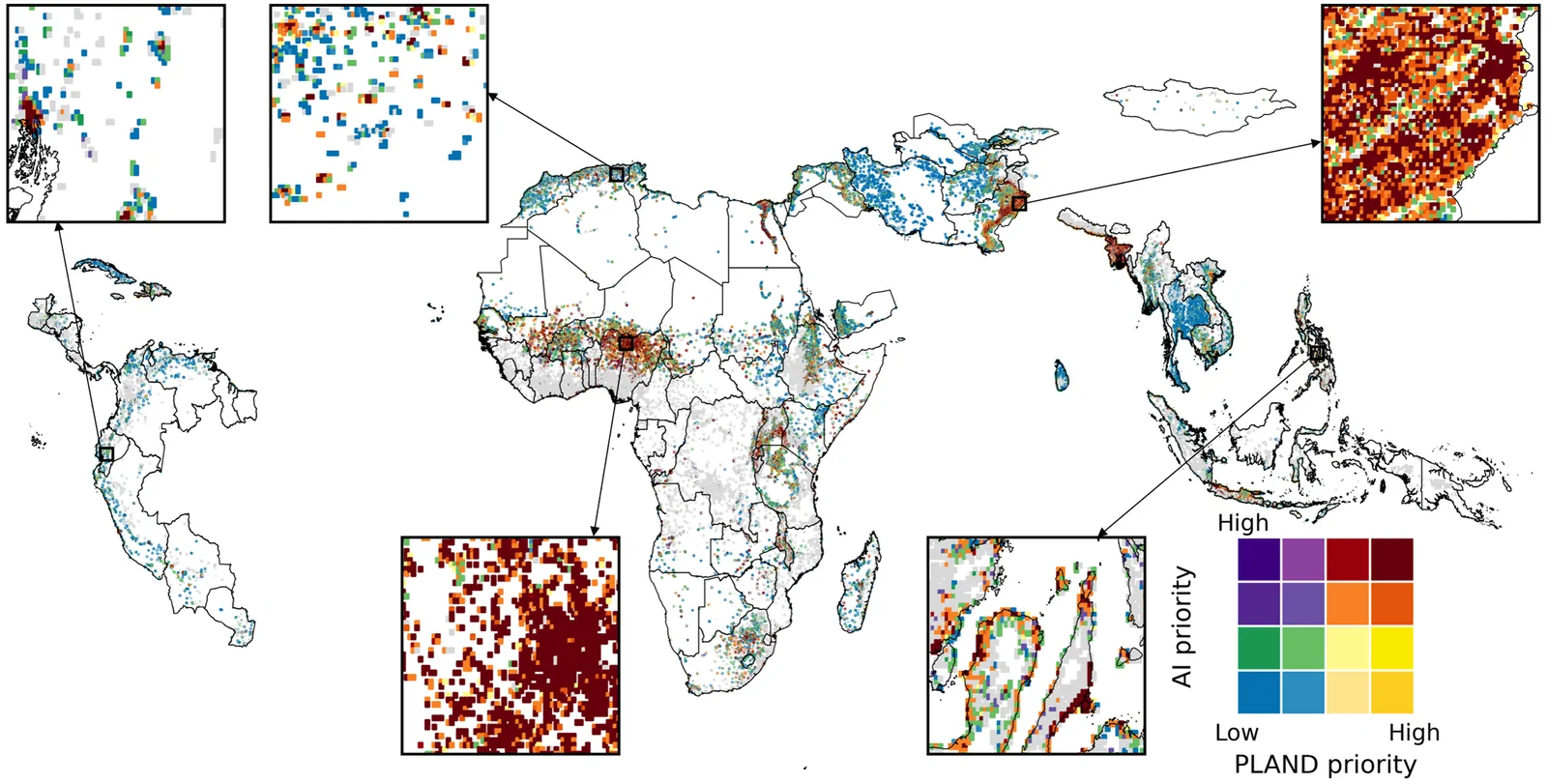
Rescaled priority rankings under counterfactual greening scenarios at the ~4 km grid level. Panels show priorities under the PLAND scenario, i.e., tree canopy expansion to the maximum feasible level capped at 30%, and the AI scenario, i.e., tree canopy aggregation optimized to the empirical 97.5th percentile conditional on canopy cover. Priority was defined based on the relative magnitude of potential health gains per square kilometer increase in tree canopy cover or per percentage-point increase in the AI, rescaled to a 0 to 1 range. Light-gray areas indicate locations with no remaining potential for tree canopy expansion or canopy aggregation optimization.

We further quantified greening priority based on the relative magnitude of potential health gains per square kilometer increase in tree canopy cover or per percentage point increase in AI, rescaled to a 0 to 1 range (*SI Appendix*, *Materials and Methods* and *Counterfactual Scenario Analysis*), to identify locations where greening interventions may yield the greatest child-health gains. The priority spots for improving PLAND and AI broadly overlap (*SI Appendix*, Fig. S15). They are concentrated in the West Sudanian Savanna, the eastern Punjab region of Pakistan, and the Ganges–Brahmaputra Delta in Bangladesh, with additional clusters observed in parts of East and Southern Africa (Fig. 4). These regions are characterized by high U5M burdens (*SI Appendix*, Fig. S12*B*) and high potential for improving tree canopy (*SI Appendix*, Fig. S13). In contrast, several high-burden regions, such as the coastal West African belt along the Gulf of Guinea (e.g., southern Nigeria and Ghana), show limited marginal gains under the modeled counterfactual greening scenario. In these areas, existing tree canopy cover is already high and exceeds the range within which the largest marginal reductions in mortality risk are observed (*SI Appendix*, Fig. S13), thereby limiting the potential for further gains from additional greening. At the national level, the highest priorities for canopy expansion were observed in Sierra Leone, Liberia, the Democratic Republic of Congo, Congo, and Somalia, while the highest priorities for improving aggregation occur in Sierra Leone, Guinea-Bissau, Liberia, Central African Republic, and Cote d’Ivoire (*SI Appendix*, Fig. S15 *C* and *D*).

## Discussion

This study presented a large-scale longitudinal analysis relating urban tree canopy cover and fragmentation to U5M at the individual level across 57 LMICs. We found that larger and more aggregated (less fragmented) tree canopies were associated with lower U5M risk within the study population. Expanding tree canopies and optimizing their aggregation may be particularly beneficial for children from lower-wealth households and those living in areas with high ambient PM_2.5_ concentrations. Using a two-lever counterfactual scenario analysis, we further identified the West Sudanian Savanna, the eastern Punjab region of Pakistan, and the Ganges–Brahmaputra Delta in Bangladesh as priority regions where canopy expansion and improved spatial configuration may be associated with the largest potential reductions in U5M under the modeled counterfactual scenarios across 98 LMICs. By integrating robust statistical associations with a counterfactual scenario framework, our findings highlight tree canopy fragmentation as a potentially modifiable feature of urban environments with potential relevance for child health under the modeled exposure–response framework. The findings are particularly salient for resource-constrained LMICs planning large-scale tree-based initiatives, where maximizing health benefits per unit of greening effort is essential.

A key contribution of this study is the longitudinal, individual-level design enabling the uncovering of robust exposure–response relationships between canopy cover, aggregation, and U5M, thereby contributing to evidence on green space exposure and child survival. Despite only one study directly linking green space exposure to U5M in the upper middle-income context (8), many cross-sectional studies have documented protective associations between green space quantity or greenness and a range of perinatal and early-life outcomes, including preterm birth, low birthweight, congenital anomalies, infections, and child development, which are major contributors to U5M (12, 49–51). In this study, we observed a nonlinear PLAND–U5M relationship, suggesting that the protective association reached a plateau at approximately 30% canopy cover and became progressively weaker at higher canopy cover levels, with the estimated mortality risk showing an upward trend thereafter. This pattern suggests that gains associated with increasing canopy cover may be most pronounced at low-to-intermediate levels. By contrast, we found a consistently protective association between canopy aggregation and U5M, independent of canopy amount. This finding is broadly consistent with previous studies, which have largely focused on adult health outcomes, including mortality (38, 39, 42), noncommunicable diseases (41), and mental health (40), as well as on preterm births (7). The linear association observed between AI and U5M mirrors patterns reported in Swiss adults on natural-cause mortality (42), reinforcing the relevance of canopy configuration for health across the life course.

Several plausible mechanisms may explain how canopy cover and, in particular, spatial aggregation influence child health outcomes. The leading causes of U5M vary across developmental stages. During the neonatal period (0 to 28 d), the predominant causes include premature birth, birth complications, and congenital anomalies (1). Among these, preterm birth is most directly linked to maternal health during pregnancy. Exposure to trees may contribute to healthier gestational environments by reducing maternal exposure to ambient air pollution, lowering extreme heat stress, and improving maternal mental well-being, thereby decreasing the risk of preterm delivery (14, 52–56). Aggregated tree canopies that form continuous and cohesive patches rather than small, isolated fragments are more effective at delivering these health-relevant ecosystem services. Such configurations provide stronger cooling effects, greater pollutant removal capacity, more stable shading, and higher-quality restorative environments than fragmented vegetation (30, 31, 37, 55). Beyond the neonatal stage, the dominant causes of U5M shift toward lower respiratory infections, malaria, and diarrhea (1). These infectious outcomes are closely linked to environmental stressors, including air pollution and elevated temperatures, both of which can be mitigated by more aggregated neighborhood-level tree canopies through microclimate regulation and air quality improvement. In addition, fragmented tree canopies tend to favor disturbance-tolerant and generalist species, which are often more competent reservoir hosts for certain pathogens, potentially increasing the relative abundance of highly competent hosts and amplifying pathogen transmission (35). Fragmented tree cover also intensifies edge effects and spatial mixing between human settlements and vector habitats, thereby elevating human exposure to disease vectors (36). These mechanisms are likely to be particularly pronounced in LMICs, where limited vector control, informal housing, and inadequate environmental management can further exacerbate human–vector contact.

Counterfactual scenario analysis has been widely used to estimate preventable mortality associated with expanding urban green space, but prior applications overwhelmingly target all-age populations and investigate only the quantity of green space (57, 58). Here we expand this literature by introducing two-lever counterfactual framework consisting of a feasible expansion of tree canopy cover subject to local land cover constraints, and a spatial optimization of canopy aggregation implemented with AI_res_, thereby enabling differentiated attribution of health gains to canopy cover and spatial aggregation. Although AI_res_ is defined as a statistical residual, it captures deviations in canopy aggregation from the level expected given canopy cover, effectively reflecting differences in how tree canopy is spatially arranged at a constant level of coverage. Higher AI_res_ values therefore correspond to more spatially cohesive and less fragmented canopy configurations. Such configurations can be influenced through planning interventions during canopy expansion, including contiguous planting along parcel boundaries, increasing street tree continuity to close canopy gaps, patch consolidation of adjacent small lots or vacant parcels, creation of linear corridors to connect isolated patches, and courtyard and school ground greening that stitches patches within dense neighborhoods (59). These strategies align with routine capital works undertaken by municipal authorities, and are consistent with the principles of environmental justice, suggesting that optimizing canopy configuration is operationally feasible even in resource-constrained urban settings, particularly in LMICs (60).

The comparison between the spatial distribution of U5M burden (*SI Appendix*, Fig. S12*B*) and greening priority (Fig. 4) reveals important equity implications. Populations experiencing the greatest health burdens are not always those who can benefit most from additional greening, as some already lie in high-coverage regimes where marginal gains are limited. This underscores the importance of context-specific strategies. While greening interventions may yield substantial health benefits in underserved regions with low to moderate canopy cover, areas with already high canopy coverage may require complementary approaches, such as improving access to healthcare or addressing other environmental risk factors, to further reduce health disparities.

This study has some limitations that must be acknowledged when interpreting the results. First, our tree exposure assignment relies on DHS cluster centroids that were randomly displaced (≤ 2 km in urban areas), which may have introduced exposure misclassification. Nevertheless, a displacement sensitivity analysis demonstrated high agreement between exposure estimates derived from original and randomly displaced locations (PLAND: r = 0.978, AI: r = 0.942; *SI Appendix*, Fig. S16), indicating that the tree canopy metrics used in this study are relatively robust to DHS geographic displacement. Second, we measured canopy presence and structure, rather than the real use of green spaces by people, and the absence of these behavioral data (e.g., time of activity, park visits) may dilute true effects. Our canopy metrics were derived from 30 m land cover products, which cannot resolve many microgreen spaces (e.g., street trees, pocket parks, courtyards). In addition, using annual composites limits the sensitivity of seasonal dynamics, especially in monsoonal or dryland climates where canopy presence and function vary across the year. Third, although we adjusted for a broad set of individual, household, and contextual covariates, unmeasured or imperfectly measured factors may still bias our estimates. As such, the counterfactual results should be interpreted as scenario-based estimates rather than precisely estimated causal effects. Finally, our greening scenarios may overestimate the achievable canopy expansion in some cities where climatic conditions have not traditionally supported extensive tree cover, and the estimated health gains should be interpreted as potential benefits under conditions where biophysical and spatial constraints can be adequately resolved. In addition, future climate change may further modify both the feasibility and effectiveness of urban greening interventions. For example, intensifying drought stress may constrain canopy survival and limit the sustainability of greening efforts, particularly in arid and semiarid LMIC settings (61). At the same time, rising temperatures and more frequent extreme heat events may alter the exposure–response relationships underlying the health benefits of tree canopy, potentially increasing the relative importance of cooling-related pathways while also shifting thresholds at which marginal benefits diminish. Climate change may also influence other pathways linking green space to health, such as vector ecology and infectious disease dynamics (62), thereby modifying the net effects of canopy configuration in different contexts. These interacting processes remain uncertain and were not explicitly modeled in this study, highlighting an important direction for future research integrating climate projections with urban greening and health impact assessments.

In conclusion, this study demonstrates that both the amount and spatial configuration of urban tree canopies may play a critical role in shaping early-life survival across LMICs. By showing that increasing canopy cover and reducing canopy fragmentation are associated with substantial and quantifiable reductions in U5M under counterfactual modeling scenarios, this study reframes urban forestry as a potentially actionable component of public health strategies, rather than solely an environmental or climate mitigation measure. As LMICs continue to urbanize rapidly, integrating canopy configuration into urban planning and restoration efforts offers a promising yet previously overlooked opportunity to advance child survival, provided it is pursued alongside comprehensive strategies addressing healthcare access, socioeconomic disparities, and other core drivers of mortality.

## Materials and Methods

### Mortality Data.

Data on all-cause U5M were obtained from the Demographic and Health Surveys (DHS) (45). We used surveys containing the Children’s Recode (KR) module to construct the under-5 children dataset. Additional variables were extracted from the surveys to characterize household living conditions, maternal characteristics, child characteristics, and fertility or birth-related factors. We also regenerated a harmonized wealth index comparable across countries. See Supporting Information for details.

### Tree Canopy Characteristics.

We calculate PLAND and AI, along with two alternative fragmentation metrics, within a 2-km buffer around each DHS urban cluster location using the global 30-m land-cover dynamic monitoring product GLC _ FCS30D. These metrics were computed using Fragstats (v4.2.64) with the 8-cell neighbor rule (46). See Supporting Information for details.

### Statistical Analyses.

We used time-varying Cox regression models to analyze the associations between tree canopy metrics and U5M risks, with age (months) as the time scale. AI is strongly correlated with PLAND, as areas with greater tree canopy cover naturally exhibit higher spatial aggregation. To disentangle the independent effect of spatial aggregation from that of canopy quantity, and vice versa, we derived a residual aggregation metric (AI_res_). This was calculated as the residual from a regression of AI on PLAND using a natural spline with two degrees of freedom ($f_{\mathrm{AI}}$), representing the deviation of aggregation from the level expected given the amount of canopy cover. Positive AI_res_ values indicate higher-than-expected aggregation (i.e., more clustered canopies), whereas negative values indicate lower-than-expected aggregation (i.e., more fragmented canopies). We examined the potential modification effects by child sex, age group, household wealth condition, climate zones, geographical region, and annual mean PM_2.5_ concentration in stratified models. We also performed several sensitivity analyses. See Supporting Information for details.

### Counterfactual Scenario Analysis.

We conducted a counterfactual scenario analysis to estimate the potential magnitude of changes in U5M in 2015 under two policy-relevant scenarios: increasing urban tree canopy cover to meet the 30% target, motivated by the observed exposure–response relationship and the 3-30-300 tree exposure guideline in the counterfactual PLAND scenario, and further optimizing tree canopy aggregation to the empirically observed upper bound (i.e., 97.5th percentile) corresponding to that canopy cover in the counterfactual AI scenario. To identify locations where greening could yield the greatest potential health gains, we quantified priority based on the efficiency of each intervention: potential mortality reduction per square kilometer increase in tree canopy area for the PLAND scenario, and per percentage point increase in aggregation for the AI scenario. These efficiency metrics were rescaled to a 0 to 1 range for mapping and comparison across grids. See Supporting Information for details.

## Supplementary Material

Appendix 01 (PDF)

## Data Availability

The health data used in this study are available from the Demographic and Health Surveys (DHS) Program (https://www.dhsprogram.com/Data/) upon reasonable request and subject to DHS approval (45). The authors do not have permission to share these data directly.
