# Supplementary material for: Urban tree canopy fragmentation and child mortality in low- and middle-income countries
Source: Proc Natl Acad Sci U S A. 2026 Aug 21;123(34):e2606669123. doi: 10.1073/pnas.2606669123 (PMC13506054; doi:10.1073/pnas.2606669123)
Supplement: Supplementary file 1 [file pnas.2606669123.sapp.pdf]

# Urban tree canopy fragmentation and child mortality in low- and middle-income countries

Dengkai Chi[1,2*], Daniel Richards[3], Gabriele Manoli[4], Jun Yang[5], John S. Ji[6], Ye Zhang[7], Brenda Lin[8], Amy Hahs[9], Congqiang Liu[1], Yue Zhu[2,10], Yeshan Qiu[2,11], Jing Wang[2], Paolo Burlando[12], Simone Fatichi[13], Puay Yok Tan[11]

[*]Corresponding author: Dengkai Chi (dengkai_chi@tju.edu.cn)

[1] School of Earth-System Science, Institute of Surface Earth System Science, Tianjin University, Tianjin, China
[2] Singapore–ETH Centre, Future Cities Laboratory Global, Singapore 138602, Singapore
[3] Bioeconomy Science Institute, Lincoln, New Zealand
[4] Laboratory of Urban and Environmental Systems, École Polytechnique Fédérale de Lausanne, Lausanne, Switzerland
[5]Department of Earth System Science, Institute for Global Change Studies, Ministry of Education Ecological Field Station for East Asian Migratory Birds, Tsinghua University, Beijing, China
[6] Vanke School of Public Health, Tsinghua University, Beijing, China
[7] School of Architecture, Tsinghua University, Beijing, China
[8] CSIRO Environment, Dutton Park, QLD, Australia
[9] School of Agriculture, Food and Ecosystem Sciences, Burnley Campus, The University of Melbourne, 500 Yarra Boulevard, Richmond, Melbourne, Victoria 3121, Australia
[10] State Key Laboratory of Internet of Things for Smart City and the Department of Arts and Design, University of Macau, Macao SAR
[11] Department of Architecture, College of Design and Engineering, National University of Singapore, Singapore 117566, Singapore
[12] Institute of Environmental Engineering, ETH Zurich, Zurich 8093, Switzerland
[13] Department of Civil and Environmental Engineering, College of Design and Engineering, National University of Singapore, Singapore 117576, Singapore

## Supporting information

### Materials and Methods

**Mortality data.** Data on all-cause under-5 mortality were obtained from the Demographic and Health Surveys (DHS). Since 1984, the DHS program has conducted nationally representative surveys approximately every five years in more than 90 LMICs. The surveys employ a two-stage sampling design: in the first stage, enumeration areas (EAs; known as clusters in DHS) are systematically selected from country-specific census files, and in the second stage, a random sample of households within each cluster is chosen. EAs are the primary sampling units defined by national census authorities and typically contain approximately 100–300 households, although their geographic extent varies across countries and according to local population density. In densely populated urban areas, EAs are generally spatially compact, whereas in lower-density settings they may cover larger geographic areas. All eligible women aged 15–49 years in the selected households were interviewed. The DHS survey protocols and questionnaires were reviewed and approved by the ICF Institutional Review Board, and all analyzed data were anonymized prior to release.

We used surveys containing the Children's Recode (KR) module to construct the under-5 children dataset. Each record in the KR file represents a birth reported by an interviewed woman within the five years preceding the survey, and includes detailed information on the pregnancy, delivery, and postnatal care. We selected KR modules corresponding to surveys from Phases 4 to 8 (1997–2025) that contained a GPS dataset and all variables required for subsequent analyses (Table S1). Cluster locations in the GPS files were recorded using handheld GPS devices and randomly displaced by up to 2 km in urban areas and up to 5-10 km in rural areas for confidentiality. Given the greater positional uncertainty in rural clusters, we restricted analyses to urban clusters. We excluded children whose mothers were interviewed before 2000 or after 2019, due to the availability of tree canopy data and the potential confounding effects of the COVID-19 pandemic. Children with survival times longer than 60 months were also excluded.

Additional variables were extracted from the surveys to characterize household living conditions (source of drinking water, toilet facility, and type of cooking fuel), maternal characteristics (age, education level, and marital status), child characteristics (sex), and fertility or birth-related factors (caesarean section, place of delivery, birth order, breastfeeding, and twin status) (Table S7). The DHS program typically constructs a household wealth index for each survey using principal component analysis based on assets and utilities, but this index is not directly comparable across countries. Therefore, following the DHS approach, we re-generated a harmonized wealth index by pooling all eligible surveys and using asset-related variables consistently available across surveys, specifically, ownership of a radio, television, refrigerator, motorcycle, car, telephone, and access to electricity, from the Household Recode (HR) files. Variables used in the original wealth index by DHS with excessive missing data were excluded to preserve sample size. Six wealth groups were then defined based on quantiles of the reconstructed index. In order to make nationally representative survey data representative of all the studied countries, we followed previous studies and weighted observations by the product of country-specific household survey weights (supplied by

DHS) and country population weights (i.e., dividing the total country female population by the surveyed female population in the specific country) (1). Population counts by country were obtained from the United Nations World Population Prospectives 2019. As above, we constructed a longitudinal dataset organized at the person-month level, including a survival indicator and excluding records with incomplete information.

**Tree canopy characteristics.** There are a large number of different landscape metrics that can be used to quantify the fragmentation of tree canopies (2). For this study, we focused on three widely applied and operationally straightforward metrics that are also relevant for urban green planning: the aggregation index (AI), the percentage of like adjacencies (PLADJ), and patch density (PD) (Table S2). The AI and PLADJ both quantify how often cells of the tree canopy cover touch each other, but they differ fundamentally in what they measure. PLADJ is a raw proportion of observed like adjacencies: it counts how many edges are shared between pixels of trees but not account for how many adjacencies could exist, and therefore cannot distinguish whether high adjacency reflects true aggregation or simply the presence of many tree pixels. In contrast, AI explicitly standardizes observed adjacencies by the maximum possible number of adjacencies that the existing tree canopy cover could achieve if it were arranged in the most aggregated way, making it a more interpretable metric of functional aggregation and fragmentation (Fig. S1). It is better suited for comparing patterns across regions and for applications in public health where the degree of spatial cohesion, rather than merely adjacency counts, is the outcome of interest (3). PD counts the number of patches per unit landscape area and does not distinguish between ecologically meaningful fragmentation such as the patch size, connectivity, and shape complexity (4).

To calculate these metrics, we use the global 30-m land-cover dynamic monitoring product GLC_FCS30D (5). Multiple forest-related land-cover classes (Table S5) were combined annually from 2000 to 2019 to generate a unified urban tree canopy layer. Throughout the manuscript, tree canopy refers to this aggregated tree-covered land class. For each year, we quantified tree canopy coverage (PLAND) and three fragmentation metrics within a 2-km buffer around each cluster location to reduce the sensitivity of exposure estimates to the spatial displacement (6). These metrics were computed using Fragstats (v4.2.64) with the 8-cell neighbor rule (4). As Fragstats requires inputs in projected coordinates, we reprojected the GLC_FCS30D imagery for each country to an appropriate country-specific coordinate reference system (Table S8). Child survival time was split by calendar year, and annual tree canopy metrics were assigned to the corresponding year of follow-up for subsequent analyses.

**Environmental data.** We derived climate regions (tropical, arid, temperate, and cold) from the global 1-km Köppen–Geiger climate classification map (7). We obtained annual mean $PM_{2.5}$ concentration data at a spatial resolution of 0.01° (SatPM2.5 (8)) and air temperature data at a spatial resolution of 30 arcsec (CHELSA-monthly (9)) and extracted the values corresponding to the grid cell containing each cluster location.

**Statistical analyses.** We used time-varying Cox regression models to analyze the associations between tree canopy metrics and U5M risks, with age (months) as the time scale. AI and PLADJ are strongly correlated with PLAND, as areas with greater tree

canopy cover naturally exhibit higher spatial aggregation. To disentangle the independent effect of spatial aggregation from that of canopy quantity, and vice versa, we derived residual aggregation metrics ($AI_{res}$ and $PLADJ_{res}$). This was calculated as the residual from a regression of AI/PLADJ on PLAND using a natural spline with two degrees of freedom ($f_{AI}/f_{PLADJ}$), representing the deviation of aggregation from the level expected given the amount of canopy cover. Positive $AI_{res}$ / $PLADJ_{res}$ values indicate higher-than-expected aggregation (i.e., more clustered canopies), whereas negative values indicate lower-than-expected aggregation (i.e., more fragmented canopies). The fragmentation metric PD remained as the original format due to the low correlation with PLAND (n = 0.01).

In the main model, we included PLAND and $AI_{res}$ to investigate their associations with U5M, whereas $PLADJ_{res}$ and PD were tested separately in sensitivity analyses (see below). We evaluated multiple model specifications that included different combinations of linear and nonlinear terms (i.e., natural splines with two or three degrees of freedom) for PLAND and $AI_{res}$, comparing their performance using the Akaike Information Criterion (AIC). The specification with a two–degree of freedom natural spline for PLAND and a linear term for $AI_{res}$ produced the lowest AIC, and was therefore selected as the final main model (Table S9). The effect measure for each tree canopy metric was the hazard ratio (HR) and 95% confidence intervals (CIs) estimated per unit increase in each metric. We adjusted for key confounders, including climate region, household wealth index, and maternal characteristics, which may influence both tree exposure and under-5 mortality. We also included the above-mentioned variables on household living conditions and fertility or birth-related factors and annual mean $PM_{2.5}$ concentration as established predictors of child health to improve estimation precision, although not all are assumed to directly affect tree exposure (Table S7). Sex of child and survey were included as strata to allow for flexible baseline functions. We used robust standard errors clustered at the individual child level to relax the assumption of independently and identically distributed error terms.

We examined the potential modification effects by child sex (male vs. female), age group (≤12 months vs. >12 months), household wealth condition (lowest three vs. highest three quintiles, based on the regenerated six-quintile wealth index), climate zones (tropical vs. non-tropical), geographical region (Sub-Saharan Africa vs. outside of Sub-Saharan Africa), and annual mean $PM_{2.5}$ concentration (above vs. below the median of 31.5 μg/m$^3$) in stratified models.

We also performed several sensitivity analyses. First, we explored the associations between U5M and the other two fragmentation metrics by replacing $AI_{res}$ with $PLADJ_{res}$ or PD in the main model. Second, because the cooling effects of urban trees are well established, temperature was considered a potential mediator rather than a confounder and was therefore not included in the primary models. In contrast, current evidence regarding the effects of urban trees on ambient air pollution is mixed and context dependent (10, 11). Accordingly, annual mean $PM_{2.5}$ concentrations were treated as a potential confounder and included in the primary models. As a sensitivity analysis, we further adjusted the primary models for annual mean temperature to assess whether the observed associations were robust to additional adjustment for this potential mediator.

Third, the main model was further adjusted for the coverage of other vegetation types within clusters (Table S5). Fourth, we additionally adjusted for healthcare accessibility, derived from the global map of motorized travel time to healthcare facilities (12). This variable was not included in the main model because it was only available for a single time point (2020) and therefore could not be aligned with the longitudinal exposure period. Fifth, the main model additionally incorporated the sampling weights. Sixth, we conducted a leave-one-country-out analysis by iteratively excluding each country to assess the robustness and generalizability of the main results across different settings. Finally, to account for the potential influence of prenatal exposure on child health, we conducted a lagged sensitivity analysis. Specifically, tree canopy metrics were shifted forward by one year, such that exposure in year t–1 was used to predict mortality risk in year t.

**Counterfactual scenario analysis.** We conducted a counterfactual scenario analysis to estimate the potential magnitude of changes in U5M under two policy-relevant scenarios: increasing urban tree canopy cover to meet the 30% target, motivated by the observed exposure-response relationship and the 3-30-300 tree exposure guideline (13) in the counterfactual PLAND scenario, and further optimizing tree canopy aggregation to the empirically observed upper bound (i.e., 97.5$^{th}$ percentile) corresponding to that canopy cover (Fig. S11) in the counterfactual AI scenario. We obtained baseline under-5 death counts at ~5 km resolution in 2015 from the Global Burden of Disease (GBD) project for 98 LMICs (14) (Supporting Information, p31). To align these data with the cluster size (i.e., 2 km radius), we first downscaled ~5 km deaths to ~1 km using population-weighted resampling based on ~1 km WorldPop age-specific under-5 population layers, and then aggregated to ~4 km by summation (Fig. S12). We restricted analyses to suburban and urban areas classified by GHSL-SMOD (15) (suburban/peri-urban, semi-dense urban cluster, dense urban cluster, urban center), yielding 184,685 grid cells.

For each grid, we computed PLAND for trees ($PLAND_{obs}$), other types of vegetation ($PLAND_{veg}$), and bare area ($PLAND_{bare}$), and AI for trees ($AI_{obs}$) for the year 2015, which served as the baseline scenario (S0). $AI_{res0}$ was computed using the regression model between AI and PLAND ($f_{AI}$) built in the association analysis:

$$AI_{res0} = AI_{obs} - f_{AI}(PLAND_{obs})$$

where $AI_{res0}$ represents the residual component of canopy aggregation after accounting for canopy amount, capturing deviations from the expected aggregation level given PLAND.

In the counterfactual PLAND scenario (S1), tree canopy coverage was increased toward the 30% target where feasible:

$$PLAND_1 = \begin{cases} \min\left(PLAND_{obs} + PLAND_{veg} + PLAND_{bare}, 30\%\right), PLAND_{obs} < 30\% \\ PLAND_{obs}, PLAND_{obs} \geq 30\% \end{cases}$$

In S1, we assumed no intervention on tree canopy aggregation and the change in $AI_1$ was due to the increase in PLAND level, but predicted AI values were constrained to the valid range of 0–100:

$$AI_{hat_1} = f_{AI}(PLAND_{obs})$$
$$AI_1 = \min(\max(AI_{hat_1} + AI_{res0}, 0), 100)$$
$$AI_{res1} = AI_1 - AI_{hat_1}$$

where $AI_{hat_1}$ denotes the expected level of canopy aggregation given $PLAND_1$.

In the counterfactual aggregation scenario (S2), we further reduced canopy fragmentation in the grids with tree canopy cover expansion (i.e., $PLAND_1 > PLAND_0$) by increasing AI to the empirically observed 97.5$^{th}$ percentile level under the corresponding PLAND value ($AI_{opt}$; Fig. S11) while constraining the predicted AI values to 0–100:

$$PLAND_2 = PLAND_1$$
$$AI_{res2} = \begin{cases} AI_{opt} - AI_{hat1}, AI_1 < AI_{opt} \\ AI_{res1}, AI_1 > AI_{opt} \end{cases}$$
$$AI_2 = \min(\max(AI_{hat1} + AI_{res2}, 0), 100)$$
$$AI_{res2} = AI_2 - AI_{hat1}$$

For each scenario, we obtained the linear predictor (lp; i.e., the log hazard) from the fitted Cox model and calculated the relative mortality change (RR) compared with baseline:

$$RR_{S1} = \exp(lp_{S1} - lp_{S0})$$
$$RR_{S2} = \exp(lp_{S2} - lp_{S0})$$

These relative changes were then applied to baseline deaths in each grid to derive scenario-based death counts under S1 and S2. The difference between baseline and counterfactual values was interpreted as the potential reduction in U5M associated with the modeled greening scenarios, under the assumption that the observed exposure–response relationships approximate underlying effects.

To identify locations where greening could yield the greatest potential health gains, we quantified priority based on the efficiency of each intervention: potential mortality reduction per square kilometer increase in tree canopy area for the PLAND scenario, and per percentage point increase in aggregation for the AI scenario. These efficiency metrics were rescaled to a 0–1 range for mapping and comparison across grids.

To quantify uncertainty in the counterfactual scenario analysis, we implemented a Monte Carlo framework that jointly propagated uncertainty from four sources: (i) the canopy aggregation model used to derive $AI_{res}$ based on PLAND, (ii) the empirical AI optimization target defined as the bin-specific 97.5$^{th}$ percentile of AI conditional on PLAND, (iii) the time-varying Cox model used to estimate the exposure–response relationships, and (iv) baseline grid-level under-5 mortality counts. First, uncertainty in the model $f_{AI}$ was incorporated by drawing regression coefficients from their estimated multivariate normal distribution and recalculating predicted AI values and residual aggregation terms for the baseline and canopy-expansion scenarios. Second, uncertainty in the empirical AI optimization target was incorporated by bootstrap resampling AI values within each PLAND bin and recalculating the bin-specific 97.5$^{th}$ percentile used in the aggregation

scenario. Third, uncertainty in the Cox model was propagated by drawing the full coefficient vector from its estimated multivariate normal distribution, preserving the covariance structure among all parameters. Finally, baseline grid-level deaths were simulated from log-normal distributions parameterized using each grid's point estimate and corresponding 95% uncertainty interval (UI), with values constrained to be positive. For each joint draw, we recomputed counterfactual mortality under the canopy expansion scenario and the subsequent aggregation-optimization scenario, and summarized the resulting grid-level and total scenario-based mortality reductions across 1,000 simulations using the 95% UI. We performed statistical analyses using packages survival v3.8.3, splines v4.5.0, ggplot2 v3.5.2 in R v4.5.0.

**Screening of DHS data**

We downloaded all available Demographic and Health Surveys (DHS) as of 4 December 2024. Records were filtered through the following steps. First, surveys with available GPS data and all required variables (Tables S7) were selected; within these surveys, children whose mothers were interviewed between 2000 and 2019 and who resided in urban clusters were retained, yielding an initial dataset of 490,020 children. Second, children with a survival time exceeding 60 months were excluded, resulting in an intermediate dataset of 489,946 children. Third, children with missing information on any covariates were excluded, reducing the dataset to 421,190 children. Finally, records with an aggregation index value of 100, arising exclusively from very low tree canopy cover, were excluded, yielding a final analytical sample of 420,244 children. The final dataset comprised 43,373 clusters across 133 surveys conducted in 57 countries (Table S1).

**Table S1. Information on the selected DHS surveys.**

Percentage of deaths was the ratio between the number of under-5 deaths and number of children included in the final analyses for each survey.

| Country | Survey | Number of children | Number of deaths | Percentage of deaths | Year of interview |
|---|---|---|---|---|---|
| Algeria | ALIR51 | 716 | 10 | 1.4 | 2008, 2009 |
| Algeria | ALIR71 | 1156 | 2 | 0.2 | 2017, 2018 |
| Angola | AOIR71 | 7788 | 332 | 4.3 | 2015, 2016 |
| Egypt | EGIR42 | 3976 | 10 | 0.3 | 2000 |
| Egypt | EGIR4A | 2004 | 47 | 2.3 | 2003 |
| Egypt | EGIR51 | 4343 | 77 | 1.8 | 2005 |
| Egypt | EGIR5A | 3678 | 89 | 2.4 | 2008 |
| Egypt | EGIR61 | 6075 | 111 | 1.8 | 2014 |
| Armenia | AMIR61 | 949 | 12 | 1.3 | 2010 |
| Armenia | AMIR72 | 969 | 7 | 0.7 | 2015, 2016 |
| Bangladesh | BDIR4J | 2010 | 120 | 6 | 2004 |
| Bangladesh | BDIR51 | 2094 | 99 | 4.7 | 2007 |
| Bangladesh | BDIR61 | 2293 | 47 | 2 | 2011 |
| Bangladesh | BDIR72 | 1421 | 16 | 1.1 | 2014 |
| Bangladesh | BDIR7R | 1565 | 27 | 1.7 | 2017, 2018 |
| Benin | BJIR41 | 1478 | 54 | 3.7 | 2001 |
| Benin | BJIR61 | 4914 | 236 | 4.8 | 2011, 2012 |
| Benin | BJIR71 | 5247 | 303 | 5.8 | 2017, 2018 |
| Bolivia | BOIR51 | 4260 | 137 | 3.2 | 2008 |
| Burkina Faso | BFIR43 | 1657 | 128 | 7.7 | 2003 |
| Burkina Faso | BFIR62 | 3112 | 183 | 5.9 | 2010 |
| Burundi | BUIR61 | 1340 | 65 | 4.9 | 2010, 2011 |
| Burundi | BUIR71 | 2078 | 92 | 4.4 | 2016, 2017 |
| Cambodia | KHIR42 | 1048 | 8 | 0.8 | 2000 |
| Cambodia | KHIR51 | 1622 | 93 | 5.7 | 2005, 2006 |
| Cambodia | KHIR61 | 2043 | 56 | 2.7 | 2010, 2011 |
| Cambodia | KHIR73 | 1877 | 26 | 1.4 | 2014 |
| Cameroon | CMIR44 | 3060 | 245 | 8 | 2004 |
| Cameroon | CMIR61 | 4571 | 287 | 6.3 | 2011 |
| Cameroon | CMIR71 | 4305 | 234 | 5.4 | 2018, 2019 |
| Central African Republic | CDIR51 | 3359 | 267 | 7.9 | 2007 |
| Central African Republic | CDIR61 | 5299 | 383 | 7.2 | 2013, 2014 |
| Chad | TDIR71 | 3762 | 345 | 9.2 | 2014, 2015 |
| Colombia | COIR61 | 9315 | 118 | 1.3 | 2009, 2010 |
| Comoros | KMIR61 | 1007 | 29 | 2.9 | 2012 |
| Côte d’Ivoire | CIIR62 | 2532 | 184 | 7.3 | 2011, 2012 |
| Dominican Republic | DRIR61 | 2545 | 54 | 2.1 | 2013 |
| Eswatini | SZIR51 | 694 | 79 | 11.4 | 2006, 2007 |

| | | | | | |
|---|---|---|---|---|---|
| Ethiopia | ETIR61 | 1644 | 84 | 5.1 | 2003 |
| Ethiopia | ETIR71 | 1954 | 66 | 3.4 | 2008 |
| Ethiopia | ETIR81 | 1327 | 71 | 5.4 | 2011 |
| Gabon | GAIR61 | 3540 | 161 | 4.5 | 2012 |
| Gabon | GAIR71 | 472 | 9 | 1.9 | 2019 |
| Ghana | GHIR4B | 1020 | 60 | 5.9 | 2003 |
| Ghana | GHIR5A | 975 | 61 | 6.3 | 2008 |
| Ghana | GHIR72 | 2341 | 112 | 4.8 | 2014 |
| Guinea | GNIR52 | 1327 | 120 | 9 | 2005 |
| Guinea | GNIR62 | 1956 | 116 | 5.9 | 2012 |
| Guinea | GNIR71 | 2229 | 111 | 5 | 2018 |
| Guinea-Bissau | GUIR71 | 4218 | 106 | 2.5 | 2014, 2015 |
| Haiti | HTIR42 | 1767 | 9 | 0.5 | 2000 |
| Haiti | HTIR52 | 2108 | 120 | 5.7 | 2005, 2006 |
| Haiti | HTIR61 | 2420 | 191 | 7.9 | 2012 |
| Haiti | HTIR71 | 1919 | 120 | 6.3 | 2016, 2017 |
| Honduras | HNIR62 | 3602 | 100 | 2.8 | 2011, 2012 |
| India | IAIR74 | 45228 | 1358 | 3 | 2015, 2016 |
| India | IAIR7E | 17009 | 335 | 2 | 2019 |
| Indonesia | IDIR42 | 5984 | 102 | 1.7 | 2002, 2003 |
| Jordan | JOIR42 | 3762 | 53 | 1.4 | 2002 |
| Jordan | JOIR51 | 6524 | 104 | 1.6 | 2007 |
| Jordan | JOIR6C | 6597 | 130 | 2 | 2012 |
| Jordan | JOIR74 | 7861 | 137 | 1.7 | 2017, 2018 |
| Kenya | KEIR52 | 1413 | 65 | 4.6 | 2008, 2009 |
| Kenya | KEIR72 | 3285 | 134 | 4.1 | 2014 |
| Kyrgyz Republic | KYIR61 | 1115 | 17 | 1.5 | 2012 |
| Lesotho | LSIR41 | 617 | 58 | 9.4 | 2004, 2005 |
| Lesotho | LSIR61 | 668 | 52 | 7.8 | 2009, 2010 |
| Lesotho | LSIR71 | 785 | 48 | 6.1 | 2014 |
| Liberia | LBIR51 | 1822 | 146 | 8 | 2006, 2007 |
| Liberia | LBIR6A | 2366 | 181 | 7.7 | 2013 |
| Liberia | LBIR7A | 1175 | 84 | 7.1 | 2019 |
| Madagascar | MDIR51 | 2145 | 93 | 4.3 | 2008, 2009 |
| Malawi | MWIR41 | 1923 | 40 | 2.1 | 2000 |
| Malawi | MWIR4E | 1131 | 74 | 6.5 | 2004, 2005 |
| Malawi | MWIR61 | 1776 | 130 | 7.3 | 2010 |
| Malawi | MWIR7A | 2762 | 110 | 4 | 2015, 2016 |
| Mali | MLIR53 | 3963 | 357 | 9 | 2006 |
| Mali | MLIR6A | 2524 | 117 | 4.6 | 2012, 2013 |
| Mali | MLIR7A | 2307 | 85 | 3.7 | 2018 |
| Mauritania | MBIR53 | 820 | 8 | 1 | 2005 |
| Morocco | MAIR43 | 2573 | 60 | 2.3 | 2003, 2004 |

| | | | | | |
|---|---|---|---|---|---|
| Mozambique | MZIR62 | 3562 | 237 | 6.7 | 2011 |
| Myanmar | MMIR71 | 1010 | 32 | 3.2 | 2015, 2016 |
| Nicaragua | NIIR61 | 2580 | 107 | 4.1 | 2012 |
| Niger | NMIR51 | 1825 | 90 | 4.9 | 2006, 2007 |
| Niger | NMIR61 | 2219 | 82 | 3.7 | 2013 |
| Nigeria | NGIR4B | 1928 | 151 | 7.8 | 2003 |
| Nigeria | NGIR53 | 7134 | 521 | 7.3 | 2008 |
| Nigeria | NGIR6A | 9687 | 580 | 6 | 2013 |
| Nigeria | NGIR7B | 11358 | 818 | 7.2 | 2018 |
| Pakistan | PKIR53 | 2898 | 161 | 5.6 | 2006, 2007 |
| Pakistan | PKIR71 | 5514 | 262 | 4.8 | 2017, 2018 |
| Peru | PEIR41 | 4832 | 21 | 0.4 | 2000 |
| Peru | PEIR51 | 5319 | 60 | 1.1 | 2003, 2008 |
| Peru | PEIR5I | 5639 | 116 | 2.1 | 2009 |
| Philippines | PHIR41 | 3196 | 66 | 2.1 | 2003 |
| Philippines | PHIR52 | 2572 | 40 | 1.6 | 2008 |
| Philippines | PHIR71 | 3254 | 70 | 2.2 | 2017 |
| Rwanda | RWIR53 | 1542 | 29 | 1.9 | 2005 |
| Rwanda | RWIR61 | 1225 | 64 | 5.2 | 2010, 2011 |
| Rwanda | RWIR70 | 1721 | 54 | 3.1 | 2014, 2015 |
| Rwanda | RWIR81 | 409 | 14 | 3.4 | 2019 |
| Senegal | SNIR4A | 3521 | 200 | 5.7 | 2005 |
| Senegal | SNIR61 | 3450 | 138 | 4 | 2010, 2011 |
| Senegal | SNIR6D | 1959 | 76 | 3.9 | 2012, 2013 |
| Senegal | SNIR7A | 1809 | 63 | 3.5 | 2015 |
| Senegal | SNIR7I | 1815 | 57 | 3.1 | 2016 |
| Senegal | SNIR7Z | 4071 | 159 | 3.9 | 2017 |
| Senegal | SNIR81 | 1884 | 58 | 3.1 | 2018 |
| Senegal | SNIR8B | 1707 | 49 | 2.9 | 2019 |
| Sierra Leone | SLIR51 | 1732 | 183 | 10.6 | 2008 |
| Sierra Leone | SLIR61 | 2803 | 251 | 9 | 2013 |
| Sierra Leone | SLIR7A | 2965 | 223 | 7.5 | 2019 |
| South Africa | ZAIR71 | 1646 | 44 | 2.7 | 2016 |
| Tajikistan | TJIR61 | 1512 | 29 | 1.9 | 2012 |
| Tajikistan | TJIR72 | 2026 | 40 | 2 | 2017 |
| Tanzania | TZIR4I | 242 | 14 | 5.8 | 2004, 2005 |
| Tanzania | TZIR63 | 1400 | 97 | 6.9 | 2009, 2010 |
| Tanzania | TZIR7B | 2392 | 149 | 6.2 | 2015, 2016 |
| The Gambia | GMIR81 | 1241 | 56 | 4.5 | 2019 |
| Timor-Leste | TLIR61 | 2155 | 70 | 3.2 | 2009, 2010 |
| Timor-Leste | TLIR71 | 1936 | 51 | 2.6 | 2016 |
| Togo | TGIR61 | 1966 | 93 | 4.7 | 2013, 2014 |
| Uganda | UGIR41 | 1369 | 30 | 2.2 | 2000, 2001 |

| | | | | | |
|---|---|---|---|---|---|
| Uganda | UGIR52 | 834 | 61 | 7.3 | 2006 |
| Uganda | UGIR61 | 1676 | 99 | 5.9 | 2011 |
| Uganda | UGIR7B | 2713 | 133 | 4.9 | 2016 |
| Zambia | ZMIR51 | 2054 | 196 | 9.5 | 2007 |
| Zambia | ZMIR61 | 4662 | 57 | 1.2 | 2013, 2014 |
| Zambia | ZMIR71 | 2962 | 147 | 5 | 2018, 2019 |
| Zimbabwe | ZWIR52 | 1316 | 76 | 5.8 | 2005, 2006 |
| Zimbabwe | ZWIR62 | 1571 | 107 | 6.8 | 2010, 2011 |
| Zimbabwe | ZWIR72 | 2315 | 100 | 4.3 | 2015 |
| | | **420,244** | **16988** | | |

**Table S2. Equations for the calculation of the tree canopy metrics in Fragstats software (v4·2·64).**

| Tree canopy metric | Equation | |
|---|---|---|
| Tree canopy coverage (PLAND; %) | $\frac{\sum_{i=1}^{n} a_i}{A} \times 100$ | $a_i$: area ($m^2$) of green patch *i*<br>A: total landscape area ($m^2$) |
| Aggregation index (AI; %) | $\frac{g}{max_g} \times 100$ | *g*: number of like adjacencies (joins) between pixels based on the single–count method<br>$max_g$: maximum number of like adjacencies (joins) between pixels based on the single–count method |
| Patch density (PD; per 100 ha) | $\frac{n}{A} \times 10000 \times 100$ | *n*: number of green patches<br>*A*: total landscape area ($m^2$) |
| The percentage of like adjacencies (PLADJ, %) | $\frac{g_{ii}}{\sum_{k}^{m} g_{ik}} \times 100$ | $g_{ii}$: number of like adjacencies (joins) between pixels of patch type (class) *i* based on the double-count method.<br>$g_{ik}$: number of adjacencies (joins) between pixels of patch types (classes) i and k based on the double-count method |

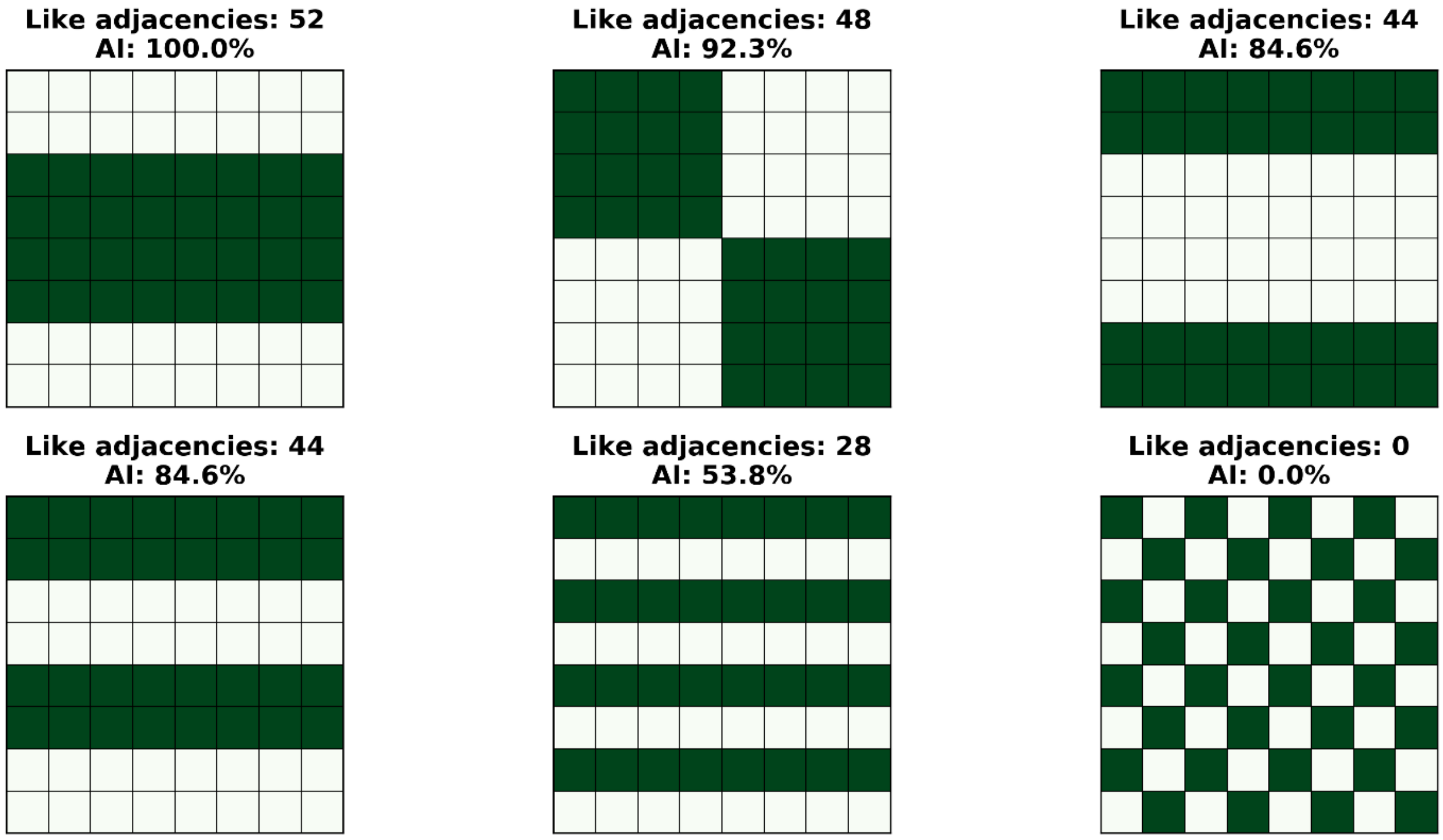


**Fig. S1. Illustrative examples of different tree canopy fragmentation levels.**

The examples have an identical total canopy area (PLAND = 50%) but varying levels of aggregation. Like adjacencies are shared edges between tree cells and the corresponding aggregation index (AI) is the ratio between the observed like adjacencies and the maximum like adjacencies (52).

**Table S3. Characteristics of tree canopy coverage (PLAND) and aggregation index (AI) and number of deaths in the whole group and subgroups.**

| | PLAND (%, mean ± SD) | AI (%, mean ± SD) | Number of deaths | Number of children |
|---|---|---|---|---|
| All | 19.9 (± 27.3) | 51.1 (± 29.4) | 16,988 | 420,244 |
| Male | 19.8 (± 27.3) | 50.9 (± 29.5) | 9,333 | 215,853 |
| Female | 20 (± 27.3) | 51.3 (± 29.4) | 7,655 | 204,391 |
| Age ≤ 12 months | 20.1 (± 27.4) | 51.3 (± 29.4) | 15,188 | 404,673 |
| Age > 12 months | 19.7 (± 27.2) | 51 (± 29.4) | 2,512 | 311,930 |
| Wealth_low (1,2,3) | 24.7 (± 29.7) | 55.4 (± 29) | 11,334 | 217,486 |
| Wealth_high (4,5,6) | 14.8 (± 23.5) | 46.6 (± 29.2) | 5,654 | 202,758 |
| Tropical | 28.5 (± 29.7) | 62.7 (± 25.1) | 10,159 | 217,885 |
| Non-tropical | 10.3 (± 20.5) | 38.3 (± 28.6) | 6,829 | 202,359 |
| Sub-Saharan Africa (SSA) | 25.7 (± 29.6) | 59 (± 27.2) | 11,854 | 208,915 |
| Non-SSA | 13.9 (± 23.3) | 43 (± 29.4) | 5,134 | 211,329 |
| $PM_{2.5}$_low (≤ 31.5 μg/m$^3$) | 24.4 (± 28.6) | 57.5 (± 27.5) | 7,780 | 210,231 |
| $PM_{2.5}$_high (> 31.5 μg/m$^3$) | 15.4 (± 25.3) | 44.8 (± 29.9) | 9,208 | 210,013 |

Note: The subgroups were divided by child sex (male, *n* = 215,853 vs. female, *n* = 204,391), age group (≤12 months, *n* = 404,673 vs. >12 months, *n* = 311,930), household wealth (lowest three, *n* = 217,486 vs. highest three quintiles *n* = 202, 758, based on the regenerated six-quintile wealth index), climate zones (tropical, *n* = 217,885 vs. non-tropical, *n* = 202,359), geographical region (SSA, *n* = 208,915 vs. non-SSA, *n* = 211,329), and annual mean $PM_{2.5}$ concentration (above, *n* = 210,231 vs. below the median of 31.5 μg/m$^3$, *n* = 210,013) for modification analyses.

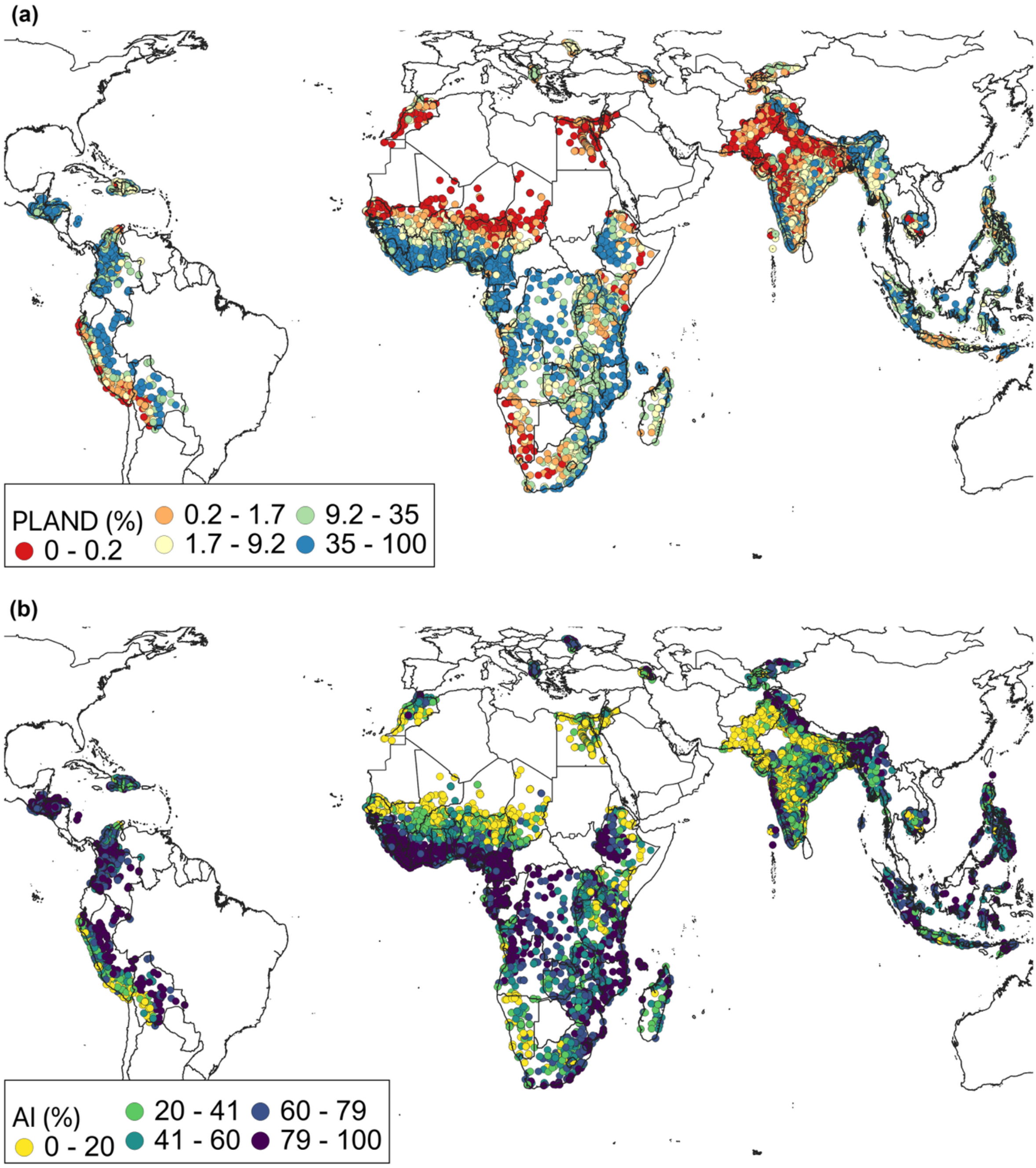


**Fig. S2. Spatial distribution of cluster-level tree canopy characteristics.**

(a) tree canopy coverage (PLAND) and (b) aggregation index (AI) averaged for the five years prior to the date of survey at the cluster level within a 2 km buffer size.

**Table S4. Hazard ratios (95% CI) for all the covariates included in the main model.**

Associations were estimated among all eligible children under five years of age (*n* = 420,244) using time-varying Cox regression models. Models were adjusted for household living conditions (drinking water source, toilet facility, and cooking fuel type), household wealth, maternal characteristics (age, education, and marital status), fertility- and birth-related factors (caesarean section, place of delivery, birth order, breastfeeding, and twin status), and environmental conditions (climate region and annual mean $PM_{2.5}$ concentration). Child sex and survey were included as strata. Effect estimates are hazard ratios with 95% confidence intervals, with robust standard errors clustered at the individual child level.

| Covariate | HR | lower .95 | upper .95 | robust se | z | Pr(>\|z\|) |
|---|---|---|---|---|---|---|
| ns(PLAND, df = 2)1 | 0.8815 | 0.7885 | 0.9854 | 0.06 | -2.22 | 0.03 |
| ns(PLAND, df = 2)2 | 1.0615 | 0.9837 | 1.1454 | 0.04 | 1.54 | 0.12 |
| AI_resid | 0.9983 | 0.9972 | 0.9995 | 0.00 | -2.69 | 0.01 |
| marital status 1 | 0.8565 | 0.7962 | 0.9214 | 0.04 | -4.16 | <0.001 |
| marital status 2 | 0.9127 | 0.8421 | 0.9892 | 0.04 | -2.22 | 0.03 |
| marital status 3 | 0.9963 | 0.862 | 1.1516 | 0.07 | -0.05 | 0.96 |
| marital status 4 | 1.2199 | 1.0729 | 1.3871 | 0.07 | 3.03 | <0.001 |
| marital status 5 | 1.1143 | 1.009 | 1.2307 | 0.05 | 2.14 | 0.03 |
| climate arid | 0.992 | 0.9325 | 1.0553 | 0.03 | -0.25 | 0.80 |
| climate temperate | 1.0607 | 1.0001 | 1.125 | 0.03 | 1.96 | 0.05 |
| climate cold | 1.4515 | 1.1212 | 1.8791 | 0.13 | 2.83 | <0.001 |
| $PM_{2.5}$ | 1.0023 | 1.001 | 1.0036 | 0.00 | 3.56 | <0.001 |
| wealth_index 2nd | 0.9325 | 0.8869 | 0.9804 | 0.03 | -2.73 | 0.01 |
| wealth_index 3rd | 0.8656 | 0.8179 | 0.916 | 0.03 | -4.99 | <0.001 |
| wealth_index 4th | 0.8156 | 0.7679 | 0.8663 | 0.03 | -6.63 | <0.001 |
| wealth_index 5th | 0.7319 | 0.6833 | 0.7839 | 0.04 | -8.91 | <0.001 |
| wealth_index 6th | 0.6969 | 0.6467 | 0.7509 | 0.04 | -9.48 | <0.001 |
| caesarian section | 1.0617 | 1.0077 | 1.1187 | 0.03 | 2.25 | 0.02 |
| education primary | 1.046 | 0.9984 | 1.0959 | 0.02 | 1.89 | 0.06 |
| education secondary | 0.8435 | 0.8027 | 0.8863 | 0.03 | -6.74 | <0.001 |
| education higher | 0.6597 | 0.6095 | 0.714 | 0.04 | -10.31 | <0.001 |
| place of delivery 2 | 0.871 | 0.8338 | 0.9099 | 0.02 | -6.19 | <0.001 |
| place of delivery 3 | 0.8075 | 0.7614 | 0.8564 | 0.03 | -7.13 | <0.001 |
| place of delivery 4 | 0.9376 | 0.7564 | 1.1623 | 0.11 | -0.59 | 0.56 |
| place of delivery 5 | 0.7474 | 0.5994 | 0.932 | 0.11 | -2.59 | 0.01 |
| age of mom | 0.9914 | 0.9882 | 0.9946 | 0.00 | -5.27 | <0.001 |
| unimproved source of water | 1.0025 | 0.9556 | 1.0516 | 0.02 | 0.10 | 0.92 |
| unimproved toilet facility | 1.0896 | 1.0454 | 1.1356 | 0.02 | 4.06 | <0.001 |
| no cooking fuel | 0.6499 | 0.3434 | 1.2301 | 0.33 | -1.32 | 0.19 |
| other cooking fuel | 1.2354 | 1.1116 | 1.373 | 0.05 | 3.92 | <0.001 |
| unclean cooking fuel | 1.0956 | 1.0394 | 1.1547 | 0.03 | 3.40 | <0.001 |
| birth order ≥ 4 | 1.1807 | 1.1302 | 1.2335 | 0.02 | 7.45 | <0.001 |
| ever breastfed | 0.0685 | 0.066 | 0.0711 | 0.02 | -140.88 | <0.001 |
| multiple birth | 3.1208 | 2.9513 | 3.3 | 0.03 | 39.95 | <0.001 |

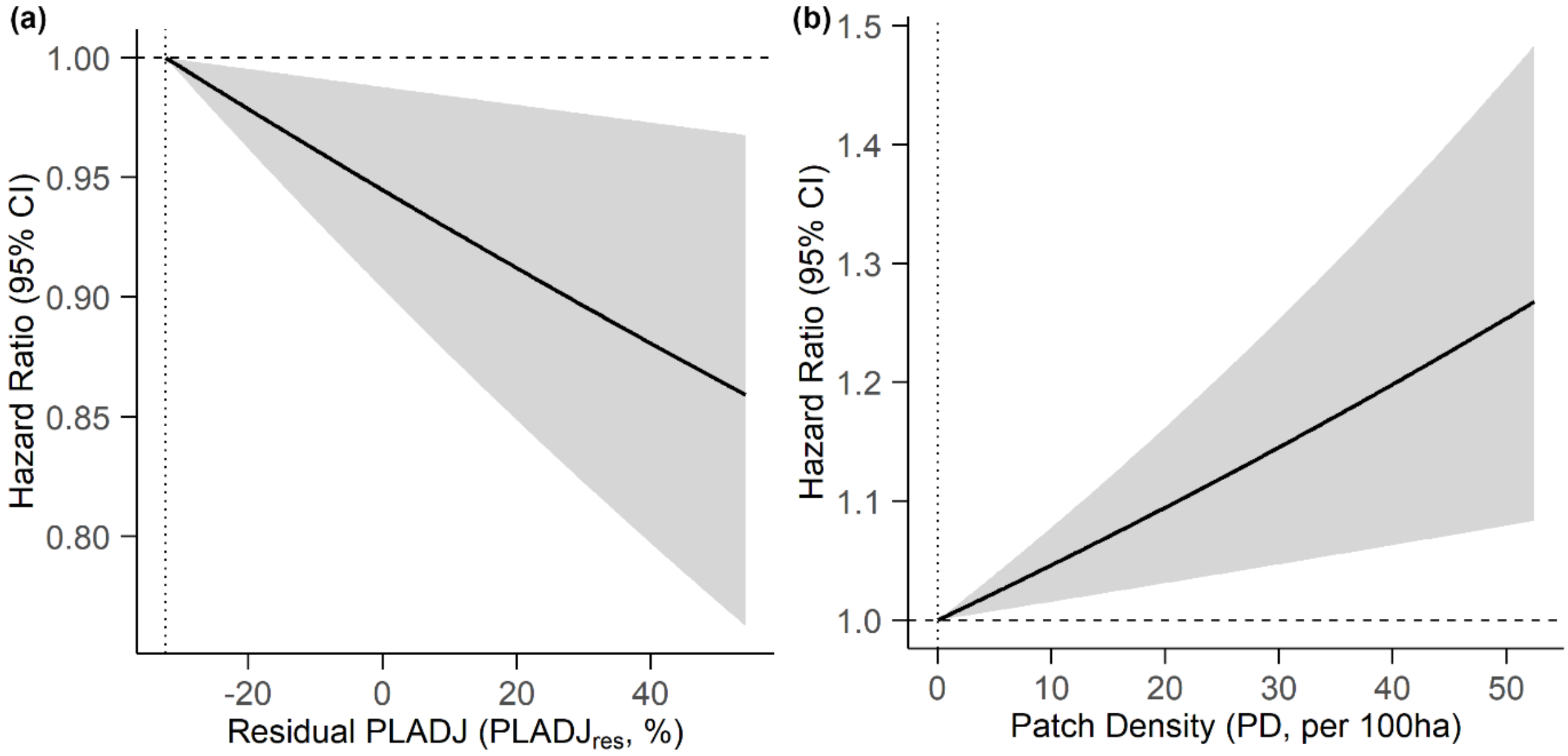


**Fig. S3. Exposure–response relationships between tree canopy fragmentation and under-5 mortality.**

Exposure–response curves for (a) the residual percentage of like adjacencies ($PLADJ_{res}$) and (b) patch density (PD) in relation to under-5 mortality. Associations were estimated among all eligible children under five years of age (*n* = 420,244) using time-varying Cox regression models. Models were adjusted for household living conditions (drinking water source, toilet facility, and cooking fuel type), household wealth, maternal characteristics (age, education, and marital status), fertility- and birth-related factors (caesarean section, place of delivery, birth order, breastfeeding, and twin status), and environmental conditions (climate region and annual mean $PM_{2.5}$ concentration). Child sex and survey were included as strata. Effect estimates are hazard ratios with 95% confidence intervals, with robust standard errors clustered at the individual child level.

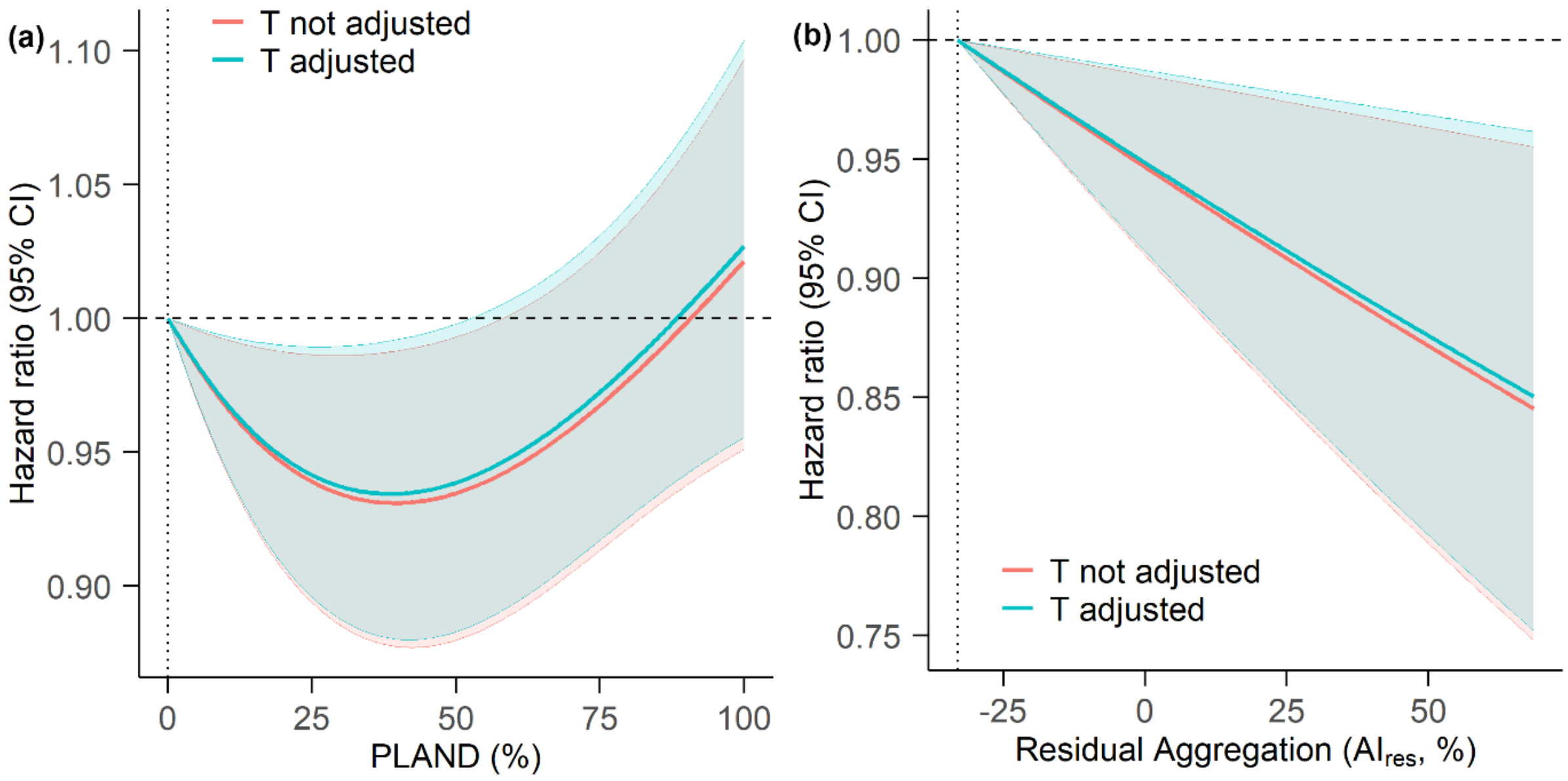


**Fig. S4. Exposure–response relationships between tree canopy and under-5 mortality after additionally adjusting for annual mean air temperatures.**

Exposure–response curves for (a) tree canopy coverage (PLAND) and (b) residual aggregation index ($AI_{res}$) in relation to under-5 mortality. Associations were estimated among all eligible children under five years of age (*n* = 420,244) using time-varying Cox regression models. Models were adjusted for household living conditions (drinking water source, toilet facility, and cooking fuel type), household wealth, maternal characteristics (age, education, and marital status), fertility- and birth-related factors (caesarean section, place of delivery, birth order, breastfeeding, and twin status), environmental conditions (climate region and annual mean $PM_{2.5}$ concentration), and annual mean air temperatures (T). Child sex and survey were included as strata. Effect estimates are hazard ratios with 95% confidence intervals, with robust standard errors clustered at the individual child level.

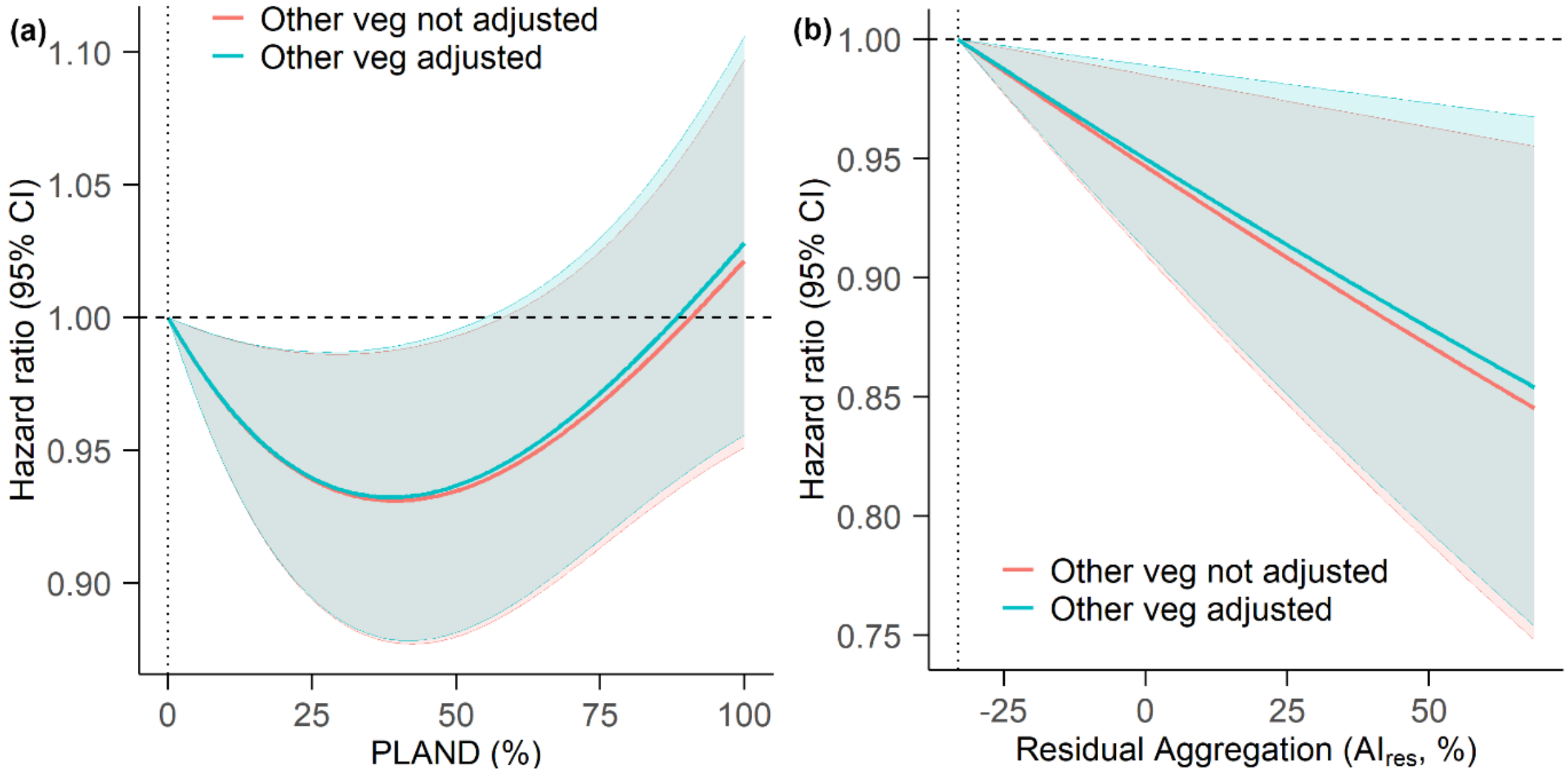


**Fig. S5. Exposure–response relationships between tree canopy and under-5 mortality after additionally adjusting for the coverage of other vegetation types (veg).**

Exposure–response curves for (a) tree canopy coverage (PLAND) and (b) residual aggregation index ($AI_{res}$) in relation to under-5 mortality. Associations were estimated among all eligible children under five years of age (*n* = 420,244) using time-varying Cox regression models. Models were adjusted for household living conditions (drinking water source, toilet facility, and cooking fuel type), household wealth, maternal characteristics (age, education, and marital status), fertility- and birth-related factors (caesarean section, place of delivery, birth order, breastfeeding, and twin status), environmental conditions (climate region and annual mean $PM_{2.5}$ concentration), and the coverage of other vegetation types (veg). Child sex and survey were included as strata. Effect estimates are hazard ratios with 95% confidence intervals, with robust standard errors clustered at the individual child level.

**Table S5. Land cover reclassification based on the global 30-m land-cover dynamic monitoring product (GLC_FCS30D).**

| **Land cover** | **Classification system in GLC_FCS30D** |
|---|---|
| Tree canopy cover | Open evergreen broadleaved forest |
| | Closed evergreen broadleaved forest |
| | Open deciduous broadleaved forest (0.15<fc<0.4) |
| | Closed deciduous broadleaved forest (fc>0.4) |
| | Open evergreen needle-leaved forest (0.15< fc <0.4) |
| | Closed evergreen needle-leaved forest (fc >0.4) |
| | Open deciduous needle-leaved forest (0.15< fc <0.4) |
| | Closed deciduous needle-leaved forest (fc >0.4) |
| | Open mixed leaf forest (broadleaved and needle-leaved) |
| | Closed mixed leaf forest (broadleaved and needle-leaved) |
| Other vegetation cover | Shrubland |
| | Evergreen shrubland |
| | Deciduous shrubland |
| | Grassland |
| | Lichens and mosses |
| | Sparse vegetation (fc<0.15) |
| | Sparse shrubland (fc<0.15) |
| | Sparse herbaceous (fc<0.15) |
| Bare areas | Bare areas |
| | Consolidated bare areas |
| | Unconsolidated bare areas |

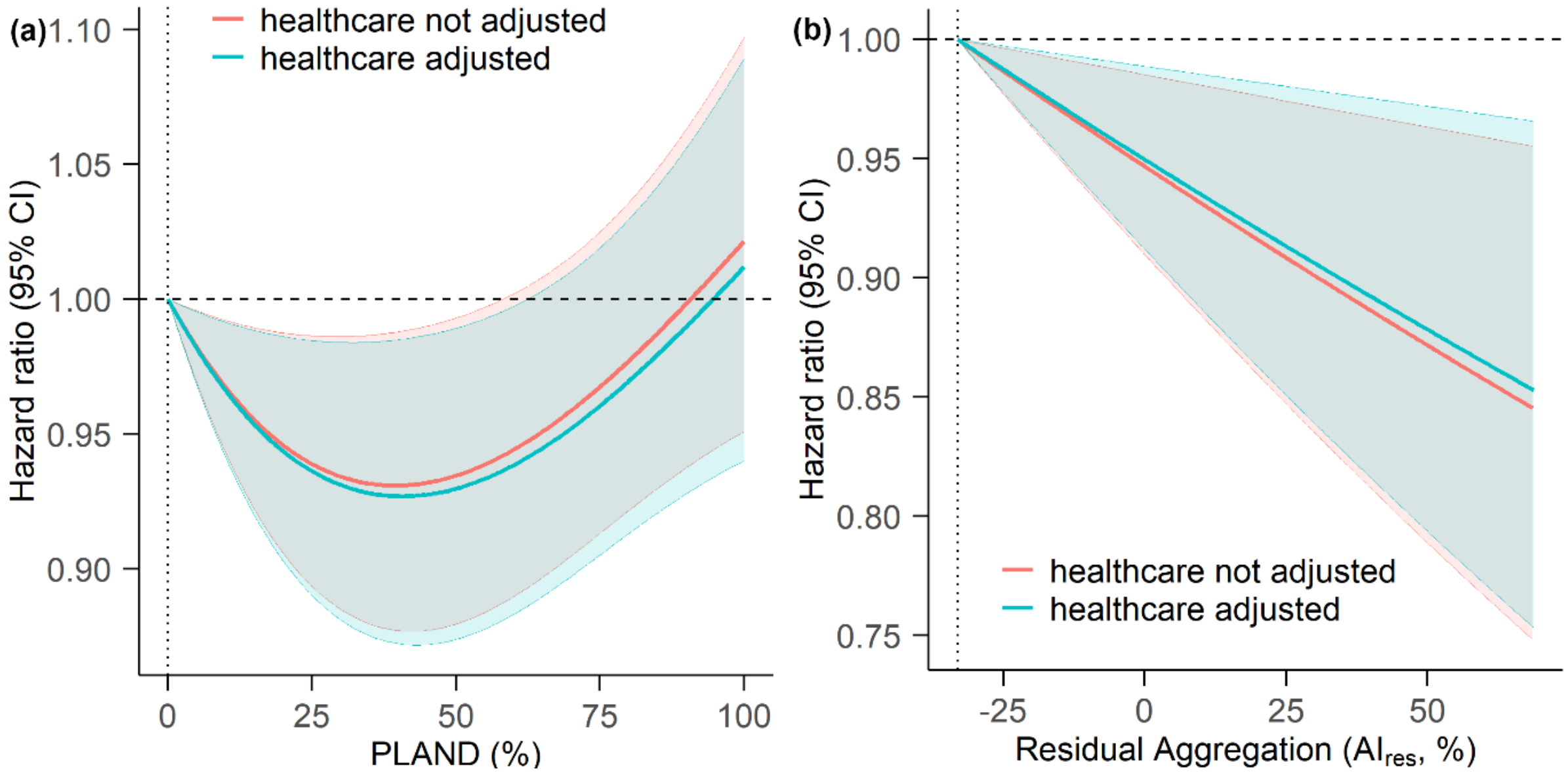


**Fig. S6. Exposure–response relationships between tree canopy and under-5 mortality after additionally adjusting for the motorized travel time to healthcare facility.**

Exposure–response curves for (a) tree canopy coverage (PLAND) and (b) residual aggregation index ($AI_{res}$) in relation to under-5 mortality. Associations were estimated among all eligible children under five years of age (*n* = 403,032) using time-varying Cox regression models. Models were adjusted for household living conditions (drinking water source, toilet facility, and cooking fuel type), household wealth, maternal characteristics (age, education, and marital status), fertility- and birth-related factors (caesarean section, place of delivery, birth order, breastfeeding, and twin status), environmental conditions (climate region and annual mean $PM_{2.5}$ concentration), and the motorized travel time to healthcare facility. Child sex and survey were included as strata. Effect estimates are hazard ratios with 95% confidence intervals, with robust standard errors clustered at the individual child level.

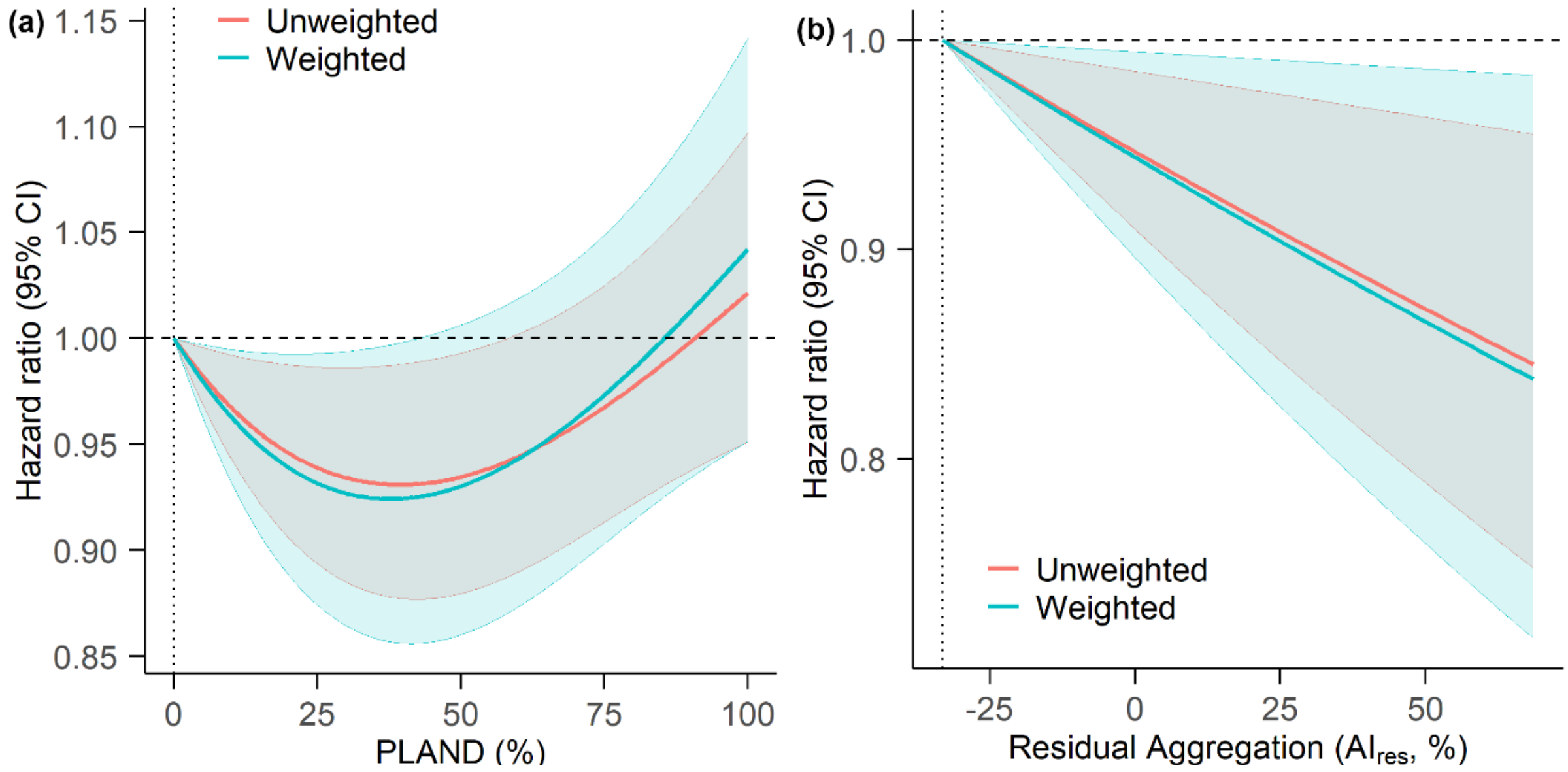


**Fig. S7. Exposure–response relationships between tree canopy and under-5 mortality after accounting for sampling weights.**

Exposure–response curves for (a) tree canopy coverage (PLAND) and (b) residual aggregation index ($AI_{res}$) in relation to under-5 mortality. Associations were estimated among all eligible children under five years of age (*n* = 419,365) using time-varying Cox regression models. Models were adjusted for household living conditions (drinking water source, toilet facility, and cooking fuel type), household wealth, maternal characteristics (age, education, and marital status), fertility- and birth-related factors (caesarean section, place of delivery, birth order, breastfeeding, and twin status), and environmental conditions (climate region and annual mean $PM_{2.5}$ concentration). Child sex and survey were included as strata. Effect estimates are hazard ratios with 95% confidence intervals, with robust standard errors clustered at the individual child level.

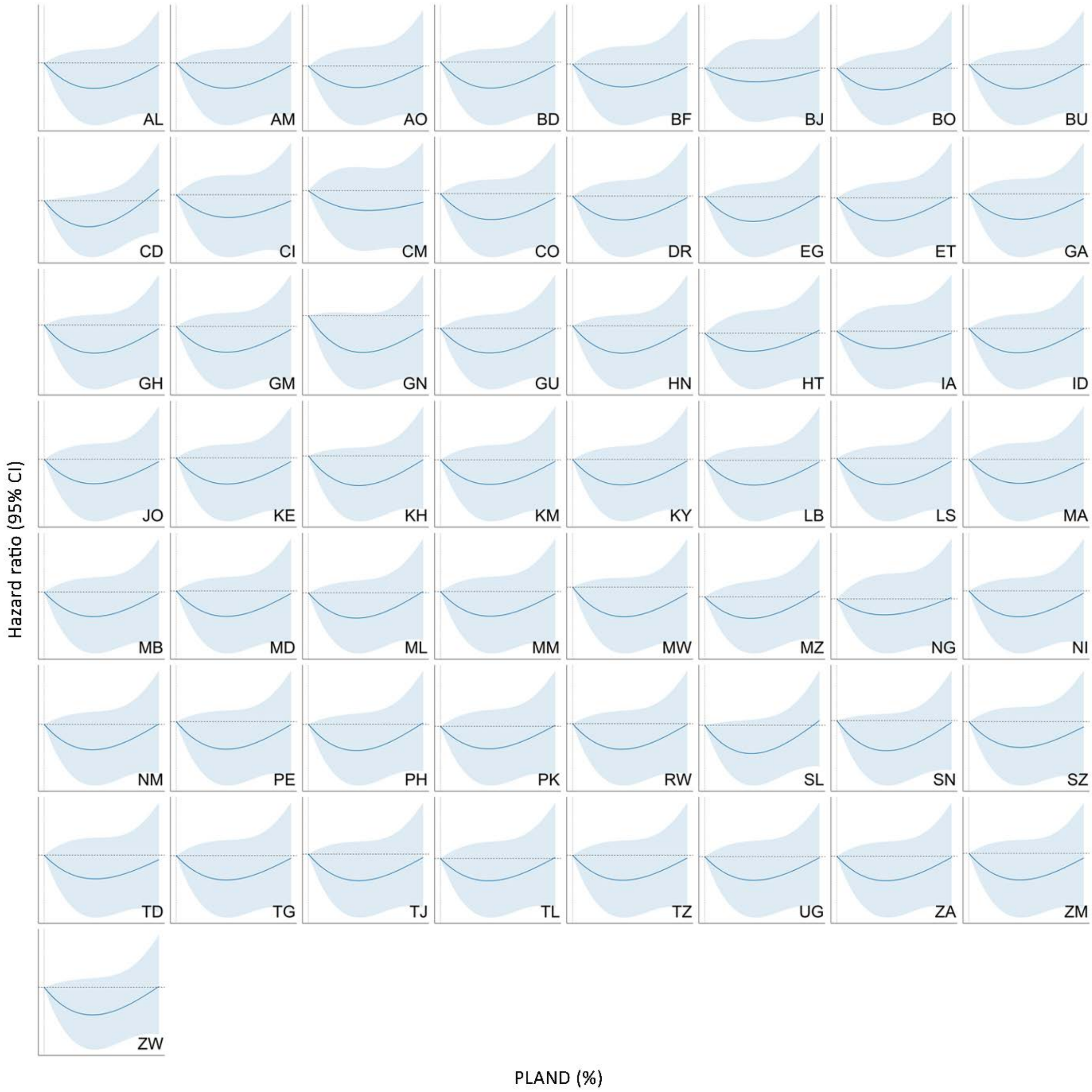


**Fig. S8. Exposure–response relationships between tree canopy coverage (PLAND) and under-5 mortality from leaving-one-country-out analysis.**

Associations were estimated using time-varying Cox regression models, sequentially excluding one country at a time to assess the robustness of the PLAND–mortality association. Models were adjusted for household living conditions (drinking water source, toilet facility, and cooking fuel type), household wealth, maternal characteristics (age, education, and marital status), fertility- and birth-related factors (caesarean section, place of delivery, birth order, breastfeeding, and twin status), environmental conditions (climate region and annual mean $PM_{2.5}$ concentration), and the motorized travel time to healthcare facility. Child sex and survey were included as strata. Effect estimates are hazard ratios with 95% confidence intervals, with robust standard errors clustered at the individual child level.

**Table S6. Hazard ratio (95% CI) for $AI_{res}$ from leaving-one-country-out analysis.**

Associations were estimated using time-varying Cox regression models, sequentially excluding one country at a time to assess the robustness of the $AI_{res}$–mortality association. Models were adjusted for household living conditions (drinking water source, toilet facility, and cooking fuel type), household wealth, maternal characteristics (age, education, and marital status), fertility- and birth-related factors (caesarean section, place of delivery, birth order, breastfeeding, and twin status), environmental conditions (climate region and annual mean $PM_{2.5}$ concentration), and the motorized travel time to healthcare facility. Child sex and survey were included as strata. Effect estimates are hazard ratios with 95% confidence intervals, with robust standard errors clustered at the individual child level.

| Country code | HR | lower .95 | upper .95 | Pr(>\|z\|) | Country code | HR | lower .95 | upper .95 | Pr(>\|z\|) |
|---|---|---|---|---|---|---|---|---|---|
| AL | 0.9983 | 0.9971 | 0.9995 | 0.01 | LB | 0.9983 | 0.9971 | 0.9995 | 0.01 |
| AM | 0.9983 | 0.9971 | 0.9995 | 0.01 | LS | 0.9983 | 0.9971 | 0.9995 | 0.01 |
| AO | 0.9985 | 0.9973 | 0.9997 | 0.01 | MA | 0.9982 | 0.997 | 0.9994 | <0.001 |
| BD | 0.9984 | 0.9972 | 0.9996 | 0.01 | MB | 0.9984 | 0.9972 | 0.9996 | 0.01 |
| BF | 0.9982 | 0.997 | 0.9994 | <0.001 | MD | 0.9983 | 0.9971 | 0.9995 | 0.01 |
| BJ | 0.9982 | 0.9969 | 0.9994 | <0.001 | ML | 0.9984 | 0.9972 | 0.9997 | 0.01 |
| BO | 0.9985 | 0.9973 | 0.9997 | 0.02 | MM | 0.9983 | 0.9971 | 0.9995 | 0.01 |
| BU | 0.9983 | 0.9971 | 0.9995 | 0.01 | MW | 0.9984 | 0.9972 | 0.9996 | 0.01 |
| CD | 0.9984 | 0.9972 | 0.9996 | 0.01 | MZ | 0.9983 | 0.9971 | 0.9995 | 0.01 |
| CI | 0.9983 | 0.9971 | 0.9995 | 0.01 | NG | 0.9988 | 0.9975 | 1.0001 | 0.07 |
| CM | 0.9987 | 0.9975 | 0.9999 | 0.03 | NI | 0.9983 | 0.9971 | 0.9995 | 0.01 |
| CO | 0.9983 | 0.9971 | 0.9995 | 0.01 | NM | 0.9984 | 0.9972 | 0.9996 | 0.01 |
| DR | 0.9983 | 0.9971 | 0.9995 | 0.01 | PE | 0.9985 | 0.9973 | 0.9997 | 0.01 |
| EG | 0.9985 | 0.9972 | 0.9997 | 0.01 | PH | 0.9984 | 0.9972 | 0.9996 | 0.01 |
| ET | 0.9983 | 0.9971 | 0.9995 | 0.01 | PK | 0.9983 | 0.9971 | 0.9996 | 0.01 |
| GA | 0.9983 | 0.9971 | 0.9995 | 0.01 | RW | 0.9984 | 0.9972 | 0.9996 | 0.01 |
| GH | 0.9984 | 0.9972 | 0.9996 | 0.01 | SL | 0.9984 | 0.9972 | 0.9996 | 0.01 |
| GM | 0.9983 | 0.9971 | 0.9995 | <0.001 | SN | 0.9984 | 0.9971 | 0.9997 | 0.01 |
| GN | 0.9985 | 0.9973 | 0.9997 | 0.02 | SZ | 0.9983 | 0.9971 | 0.9995 | 0.01 |
| GU | 0.9984 | 0.9972 | 0.9996 | 0.01 | TD | 0.998 | 0.9968 | 0.9992 | <0.001 |
| HN | 0.9984 | 0.9972 | 0.9996 | 0.01 | TG | 0.9983 | 0.9971 | 0.9995 | 0.01 |
| HT | 0.9986 | 0.9973 | 0.9998 | 0.02 | TJ | 0.9983 | 0.9971 | 0.9995 | 0.01 |
| IA | 0.9979 | 0.9967 | 0.9992 | <0.001 | TL | 0.9983 | 0.9971 | 0.9995 | 0.01 |
| ID | 0.9983 | 0.9971 | 0.9995 | 0.01 | TZ | 0.9984 | 0.9972 | 0.9996 | 0.01 |
| JO | 0.9982 | 0.9969 | 0.9994 | <0.001 | UG | 0.9984 | 0.9972 | 0.9996 | 0.01 |
| KE | 0.9983 | 0.9971 | 0.9995 | 0.01 | ZA | 0.9984 | 0.9972 | 0.9996 | 0.01 |
| KH | 0.9981 | 0.9969 | 0.9993 | <0.001 | ZM | 0.9984 | 0.9972 | 0.9996 | 0.01 |
| KM | 0.9983 | 0.9971 | 0.9995 | 0.01 | ZW | 0.9985 | 0.9973 | 0.9997 | 0.01 |
| KY | 0.9984 | 0.9972 | 0.9996 | 0.01 | | | | | |

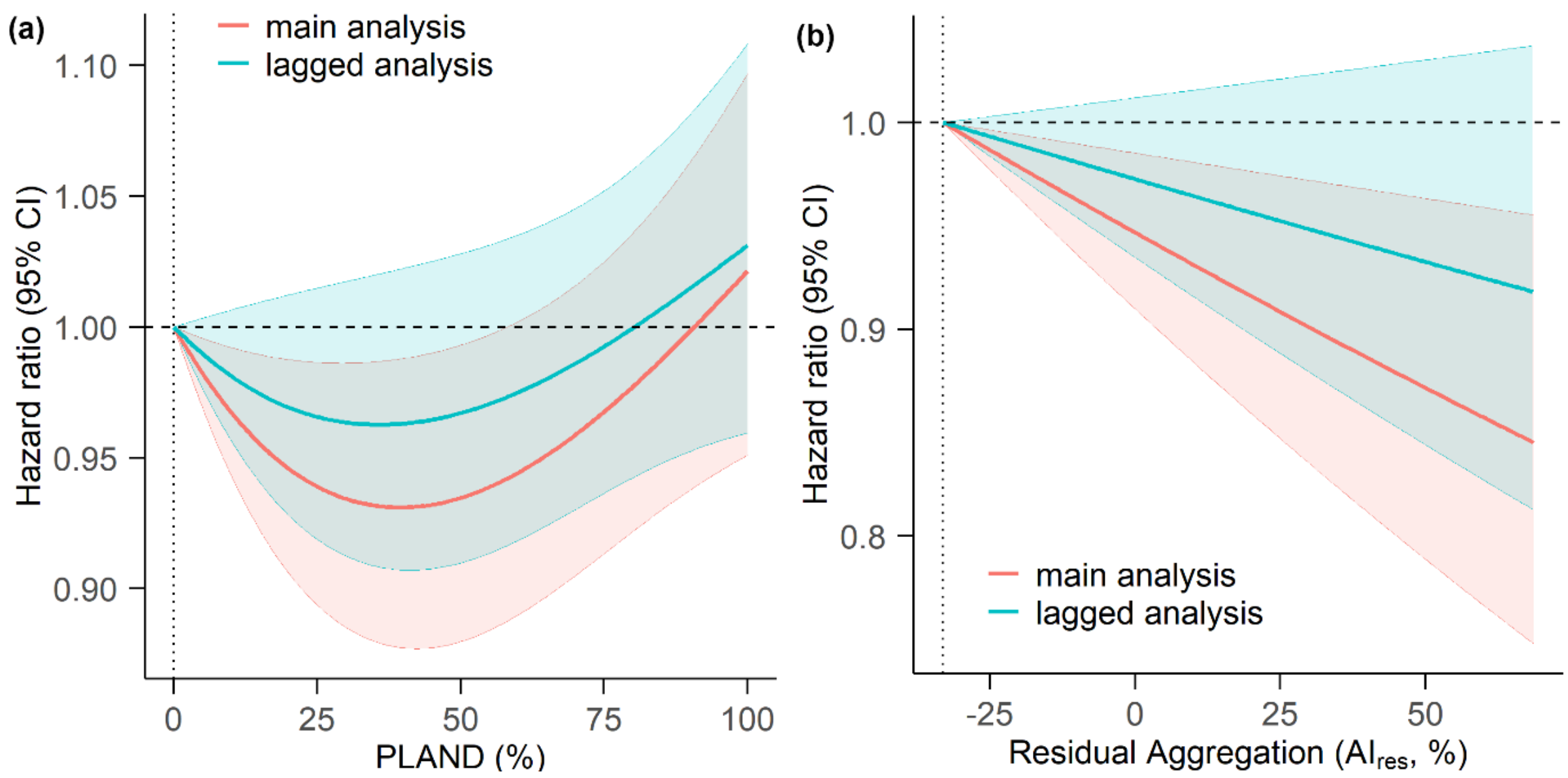


**Fig. S9. Exposure–response relationships between tree canopy and under-5 mortality in lagged exposure analysis.**

Exposure–response curves for (a) tree canopy coverage (PLAND) and (b) residual aggregation index ($AI_{res}$) in relation to under-5 mortality. In this lagged analysis, tree canopy exposure was assigned using measurements from the preceding year to incorporate pre-outcome (including prenatal and early-life) environmental conditions. Associations were estimated among all eligible children under five years of age (*n* = 404,731) using time-varying Cox regression models. Models were adjusted for household living conditions (drinking water source, toilet facility, and cooking fuel type), household wealth, maternal characteristics (age, education, and marital status), fertility- and birth-related factors (caesarean section, place of delivery, birth order, breastfeeding, and twin status), environmental conditions (climate region and annual mean $PM_{2.5}$ concentration), and the motorized travel time to healthcare facility. Child sex and survey were included as strata. Effect estimates are hazard ratios with 95% confidence intervals, with robust standard errors clustered at the individual child level.

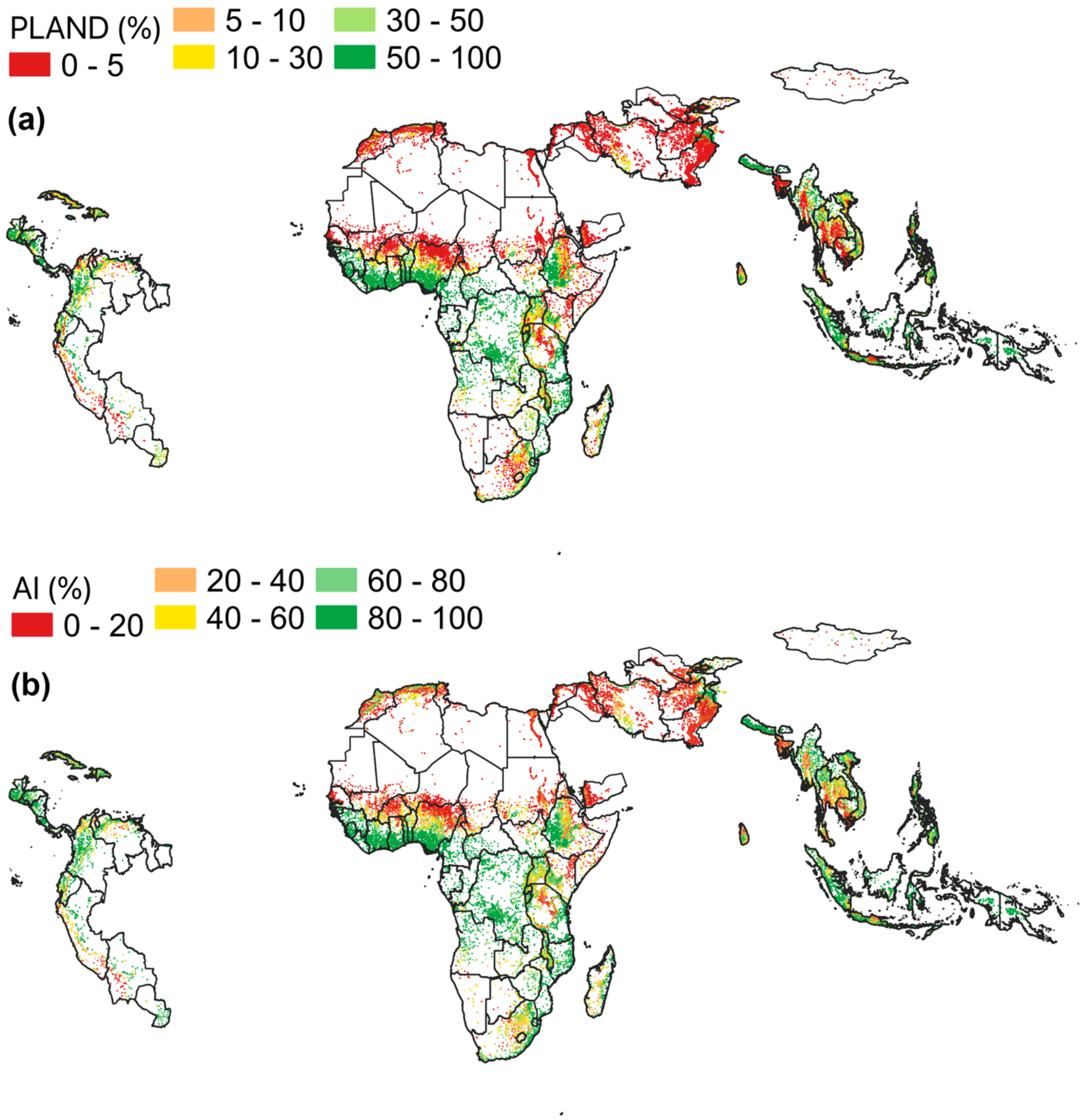


**Fig. S10. Spatial distribution of grid-level tree canopy characteristics in the counterfactual scenario analysis.**

The tree canopy coverage (PLAND) and aggregation index (AI) at the grid level (~4 km) in urban and peri-urban areas across 98 low- and middle-incomes countries in 2015.

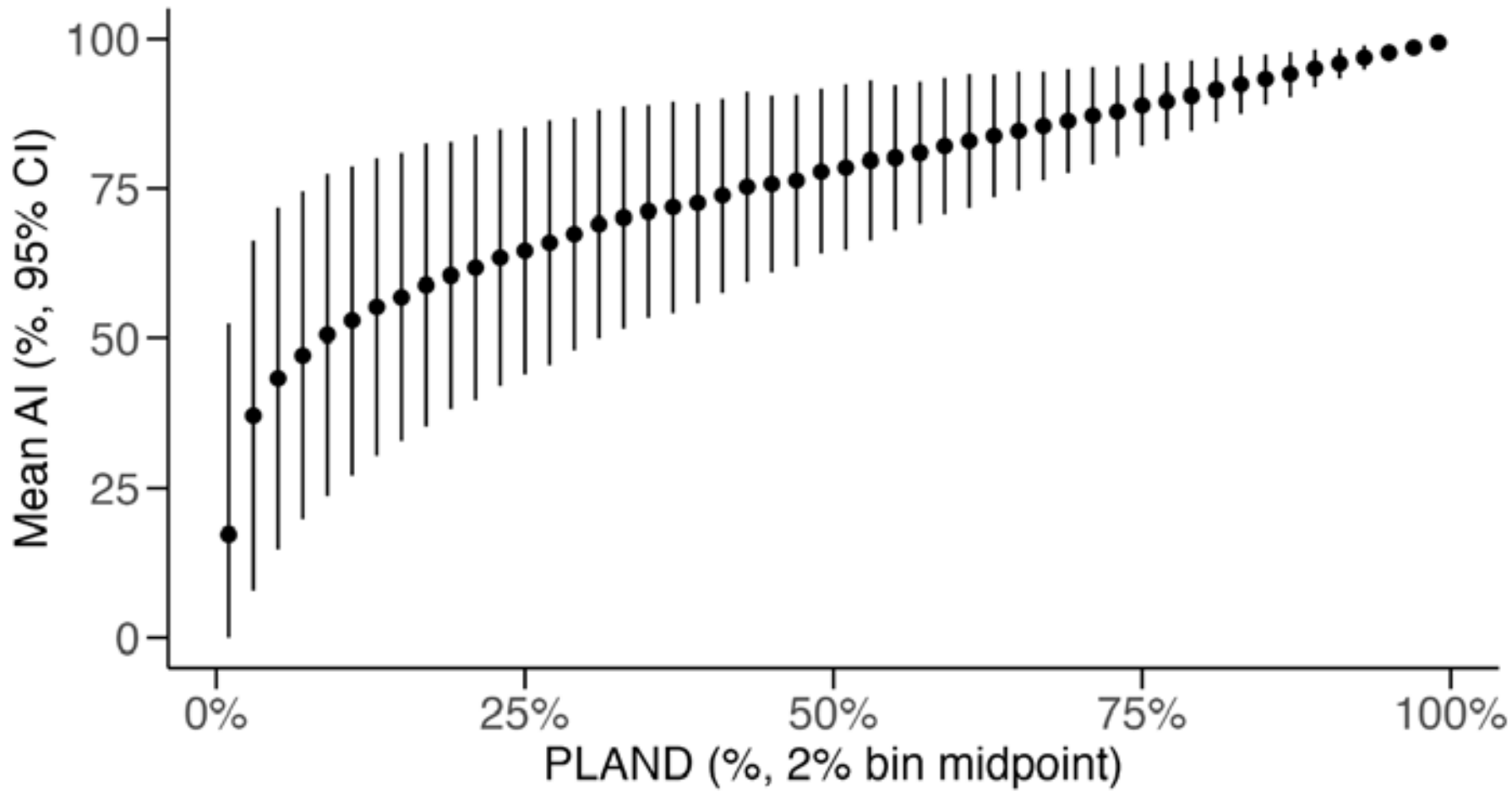


**Fig. S11. Lower (2.5th percentile) and upper (97.5th percentile) bounds of the aggregation index (AI) across levels of tree canopy coverage (PLAND) in 2% intervals.**

Bounds were derived from 184,685 urban and peri-urban ~4 km grid cells across 98 low- and middle-income countries in 2015.

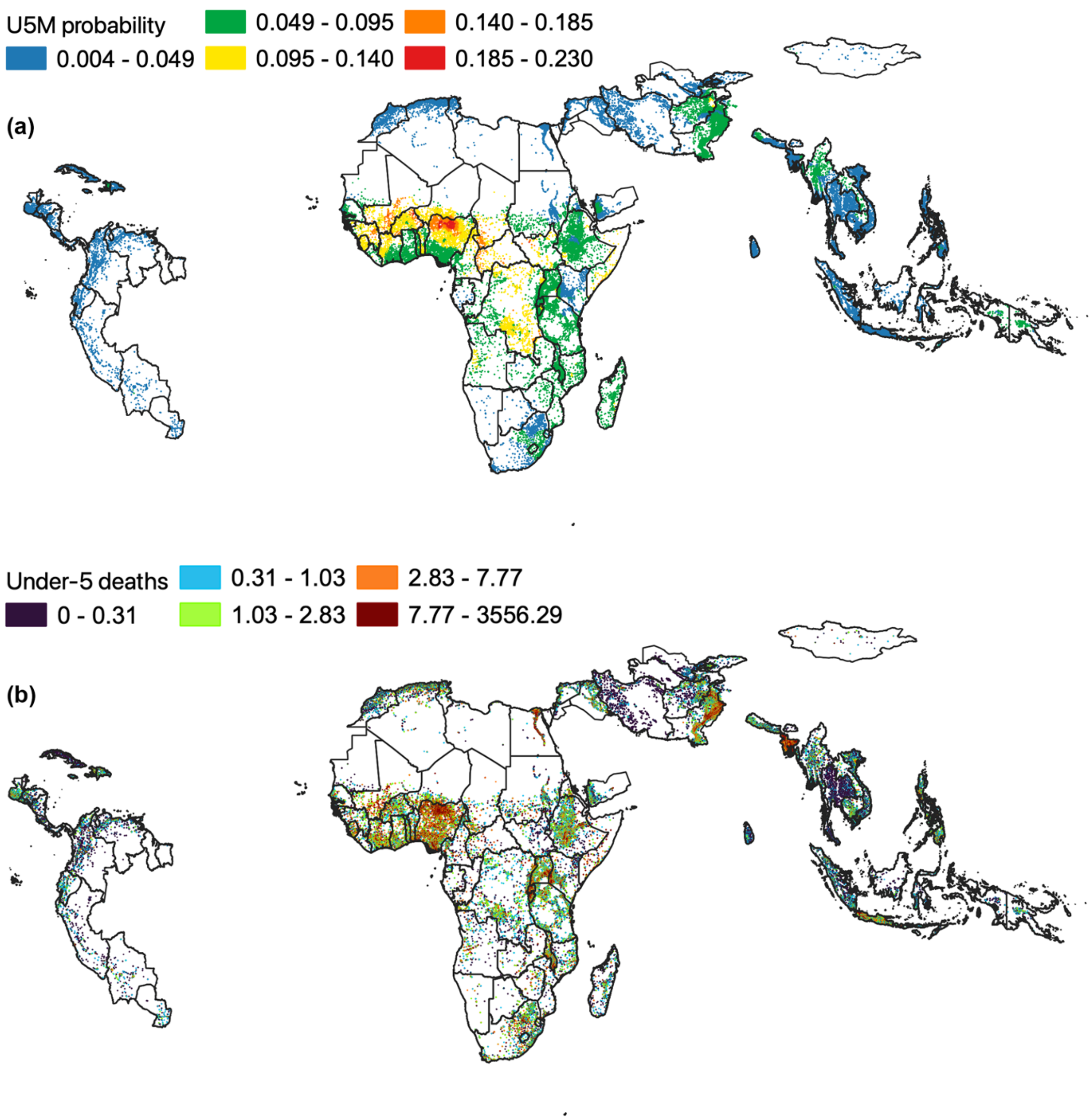


**Fig. S12. The baseline possibility and number of under-5 mortality.**

The maps have a spatial resolution of ~5 km in urban and peri-urban areas across 98 low- and middle-income countries, derived from Global Burden of Disease (GBD) project.

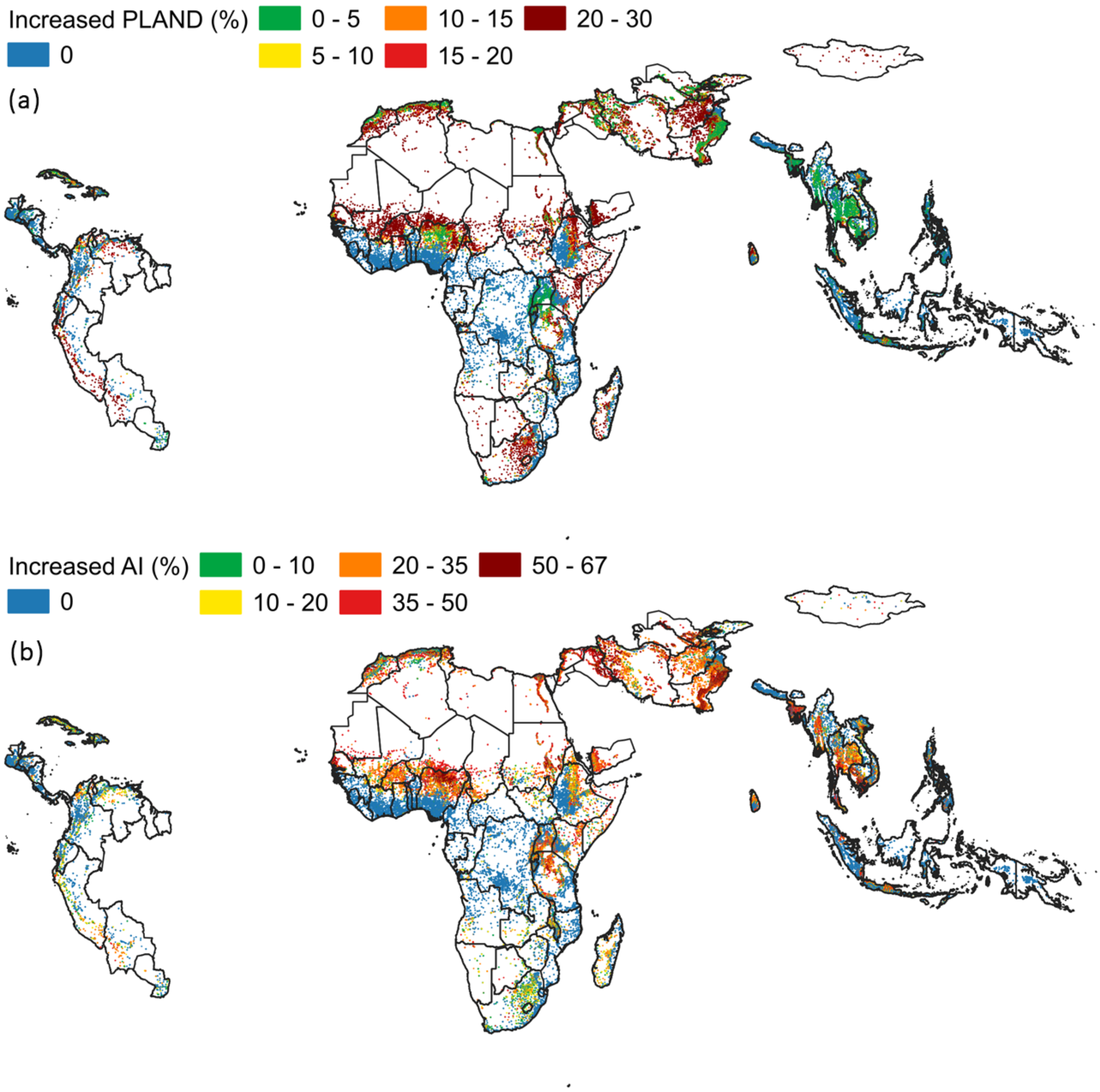


**Fig. S13. The increased level of tree canopy coverage (PLAND) and aggregation index (AI) in the counterfactual scenarios.**

The counterfactual scenarios are: (i) the PLAND scenario, in which tree canopy cover is expanded to the maximum feasible level capped at 30%, and (ii) the AI scenario, in which tree canopy aggregation is optimized to the empirical 97.5th percentile conditional on the corresponding PLAND level.

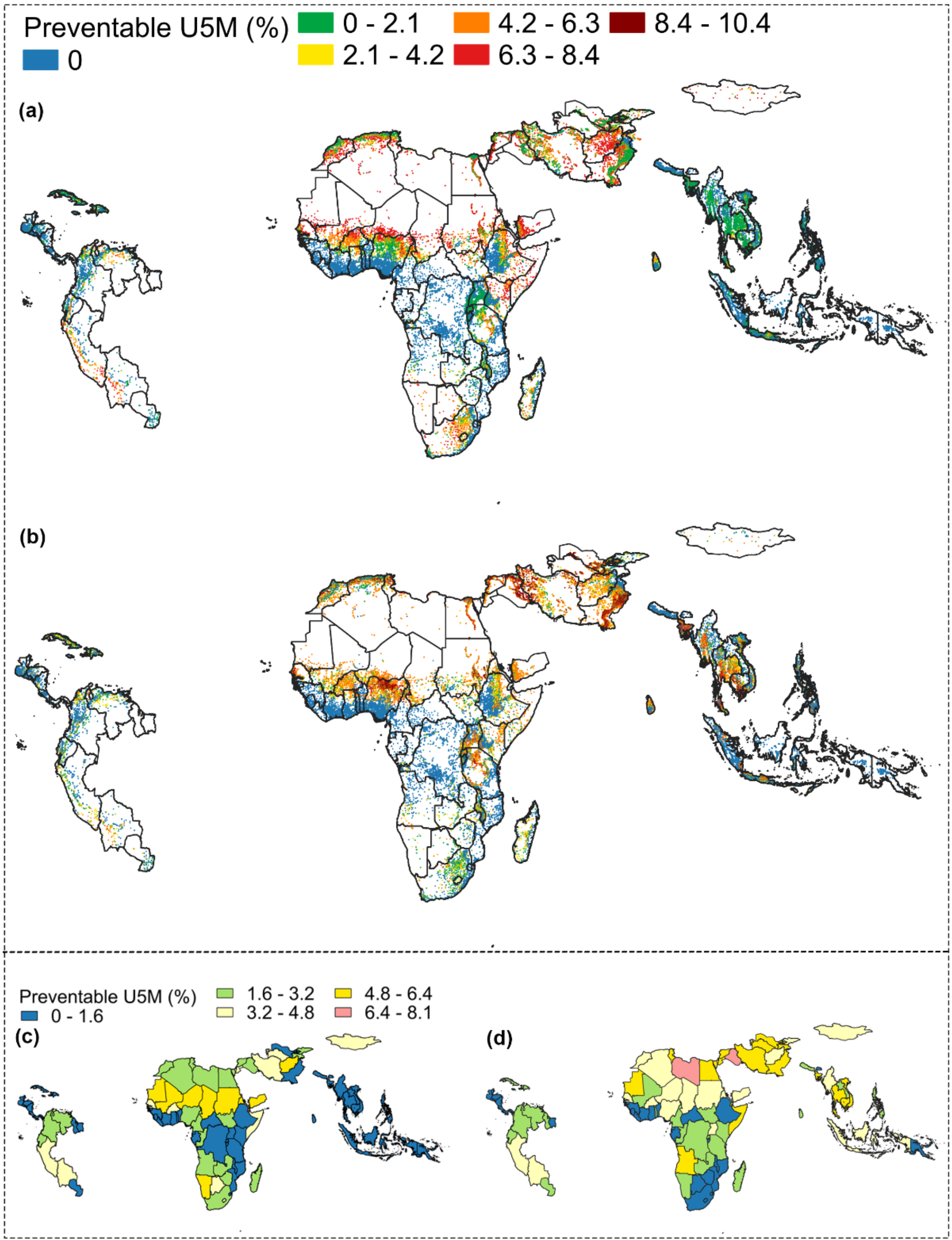


**Fig. S14. Preventable under-5 mortality associated with tree canopy expansion and aggregation in the counterfactual scenarios.**

Preventable under-5 mortality rates associated with tree canopy expansion in the counterfactual PLAND scenario, defined as expansion to the maximum feasible level capped at 30%, shown at the ~4 km grid level (a, $n$ = 185,496) and national level (c, $n$ = 98), and associated with tree canopy aggregation in the counterfactual AI scenario, defined as optimization to the 97.5$^{th}$ percentile conditional on canopy coverage, shown at the ~4 km grid level (b, $n$ = 185,496) and national level (d, $n$ = 98). Preventable mortality rates were derived from the exposure-response relationships between PLAND, $AI_{res}$, and under-5 mortality and calculated as deaths averted divided by baseline under-5 deaths (%).

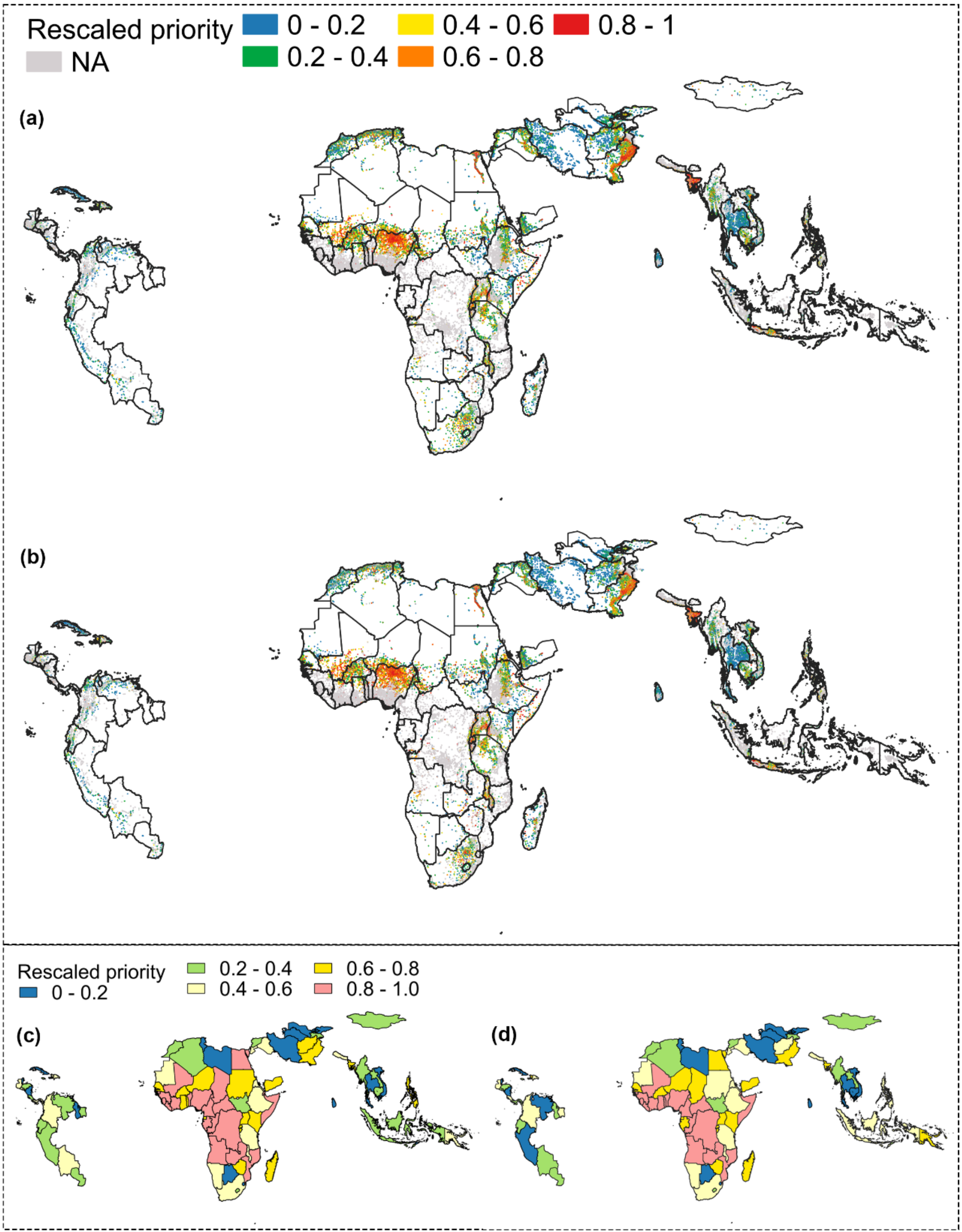


**Fig. S15. Rescaled priority rankings under counterfactual scenarios.**

Panels show priorities under the PLAND scenario, i.e., tree canopy expansion to the maximum feasible level capped at 30%, at the ~4 km grid level (a) and national level (c), and under the AI scenario, i.e., tree canopy aggregation optimized to the 97.5$^{th}$ percentile conditional on canopy cove, at the ~4 km grid level (b) and national level (d). Priority was defined as the number of preventable under-5 deaths per square kilometer increase in tree canopy cover or per percentage-point increase in the aggregation index, rescaled to a 0–1 range. Light-grey areas indicate locations with no remaining potential for tree canopy expansion or canopy aggregation optimization.

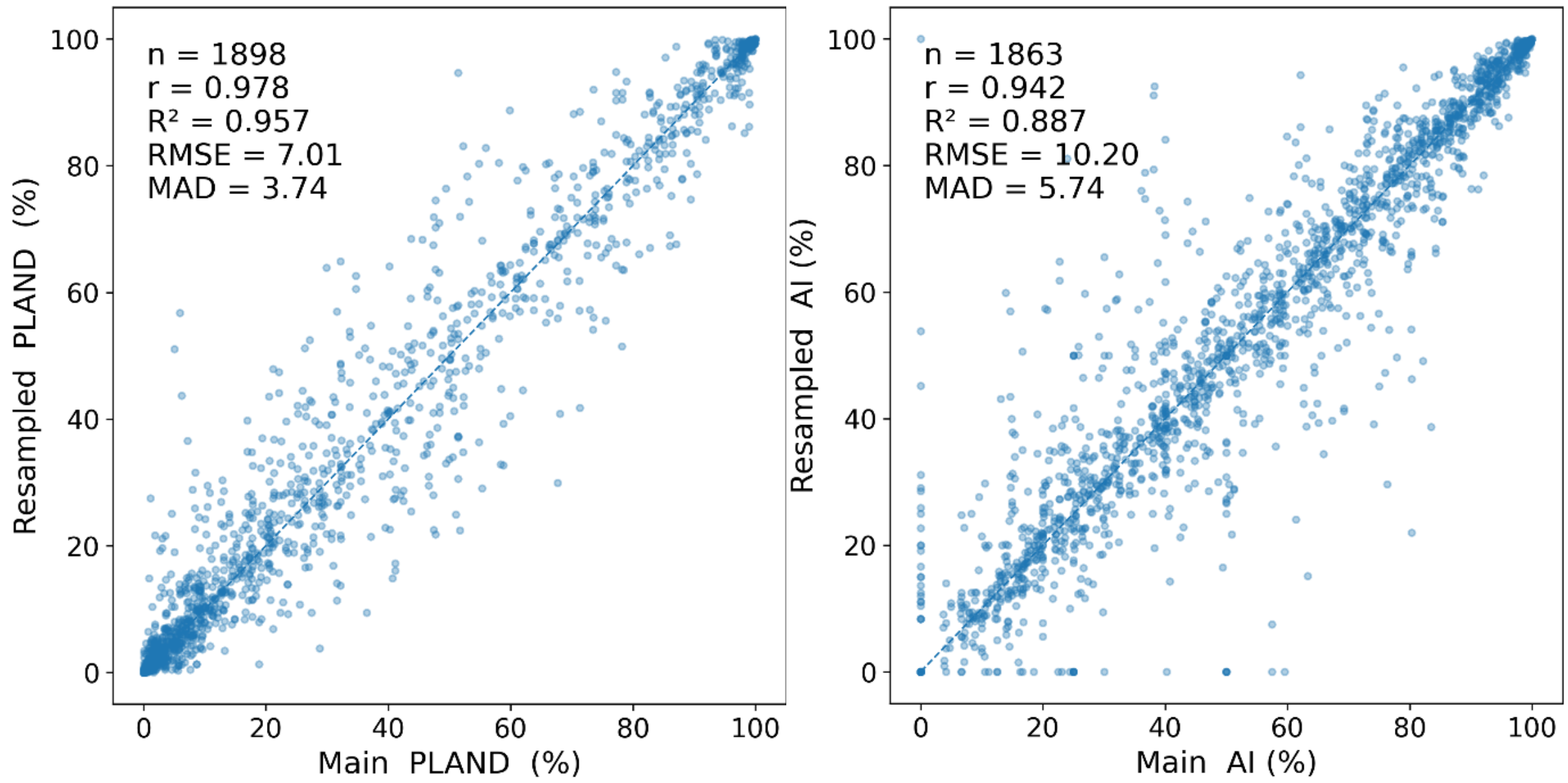


**Fig. S16. Sensitivity analysis assessing the impact of DHS geographic displacement on tree canopy exposure metrics.**

Scatterplots comparing tree canopy metrics calculated using the released DHS cluster coordinates (Main) and randomly displaced coordinates (Resampled). To evaluate the potential influence of the DHS geographic displacement procedure on exposure assessment, 1,000 urban DHS clusters were selected through proportional random sampling across countries. For each cluster, two hypothetical displaced locations were generated within a 2-km radius of the released cluster centroid, corresponding to the maximum displacement distance applied to urban DHS clusters. Tree canopy metrics were recalculated within 2-km buffers centered on the original and displaced locations using the same procedures applied in the main analysis. The displaced samples were pooled and compared with the corresponding estimates derived from the released DHS coordinates. PLAND: tree canopy coverage; AI: Aggregation Index; *n*: number of valid paired observations; *r*: Pearson correlation coefficient; $R^2$: coefficient of determination; RMSE: root mean square error; MAD: mean absolute difference.

**Table S7. Original variables from the Children's Recode (KR) and Household Recode (HR) files included in the analysis, and their corresponding recoded covariates used in the models.**

| Original variables | Explanation | Covariates | Recode |
|---|---|---|---|
| B0 | Twin code gives an order number for each child of a multiple birth | twin code | multiple birth: 1-4; single birth: 0 |
| B1 | Month of birth of child | | |
| B2 | Year of birth of child | | |
| B3 | Century month code for the date of birth of the child | | |
| B4 | Sex of child | child sex | male: 0; female: 1 |
| B5 | Whether child was alive or dead at the time of interview | | |
| B6 | Age at death of the child as reported in the questionnaire | | |
| B7 | Age at death of the child in completed months | | |
| BORD | Birth order number gives the order in which the children were born | birth order | <4; >=4 |
| M15 | Place of delivery of the child | place of delivery | home: 11-13; public facility: 21-28; private facility: 31-47; other: 51, 95, 96; missing: others |
| M17 | Whether child was born by caesarean section | caesarean section | yes: 1; no: 0; missing: others |
| M4 | The duration of breastfeeding of the child in months | breastfed status | ever breastfed: 1-90, 93, 95; never breastfed: 0, 94; missing: others |
| V000 | Alphabetic country code | | |
| V001 | Cluster number | | |
| V002 | Household number | | |
| V007 | Year of the individual review | | |

| | | | |
|---|---|---|---|
| V008 | Century month code of date of interview | | |
| V012 | Maternal age at interview | maternal age | |
| V025 | Type of place of residence | | |
| V106 | Highest education level attended | education level | no education: 0; primary: 1; secondary: 2; higher: 3; missing: others |
| V113 | Main source of drinking water for members of the household. | source of drinking water | improved: 11,12,13,14,21,31,41,51,61,62,71; unimproved: others |
| V116 | Type of toilet facility in the household. | toilet facility | improved: 11,12,13,21,22,41; unimproved: others |
| V161 | Type of cooking fuel | cooking fuel | clean: 1-4; unclean: 5-13; no food cooked in household: 88, 95; other fuels: others |
| V501 | Current marital status of the respondent | marital status | Never married nor lived with a man/woman as if married: 0; Married: 1; Not married but living with a man/woman as if married: 2; Widowed from a marriage or union and not remarried or not living with a man/woman as if married: 3; Divorced from a legal or formal marriage and not remarried or living with a man/woman as if married: 4; Separated from a marriage or consensual union: 5; |
| HV206 | Whether the household has Electricity | | |
| HV207 | Whether the household has A radio | | |
| HV208 | Whether the household has A television | | |
| HV209 | Whether the household has A refrigerator | wealth index | six quintiles after the principle component analysis for these variables |
| HV211 | Whether the household has A motorcycle | | |
| HV212 | Whether the household has A car | | |
| HV221 | Whether the household has a telephone | | |

**Table S8. EPSG codes of the projected coordinate reference systems used for each country to convert tree canopy data from geographic to projected coordinates.**

| Country | Projection | Country | Projection |
|---|---|---|---|
| Albania | EPSG:32634 | Liberia | EPSG:32629 |
| Armenia | EPSG:32638 | Lesotho | EPSG:32635 |
| Angola | EPSG:32733 | Morocco | EPSG:32629 |
| Bangladesh | EPSG:32645 | Moldova | EPSG:32635 |
| Burkina Faso | EPSG:32630 | Madagascar | EPSG:32738 |
| Benin | EPSG:32631 | Mali | EPSG:32630 |
| Bolivia | EPSG:32720 | Myanmar | EPSG:32647 |
| Burundi | EPSG:32736 | Malawi | EPSG:32736 |
| Democratic Republic of the Congo | EPSG:32734 | Mozambique | EPSG:32736 |
| Côte d'Ivoire | EPSG:32630 | Nigeria | EPSG:32632 |
| Cameroon | EPSG:32632 | Niger | EPSG:32632 |
| Colombia | EPSG:32718 | Namibia | EPSG:32733 |
| Dominican Republic | EPSG:32619 | Peru | EPSG:32718 |
| Egypt | EPSG:32636 | Philippines | EPSG:32651 |
| Ethiopia | EPSG:32637 | Pakistan | EPSG:32642 |
| Gabon | EPSG:32732 | Rwanda | EPSG:32736 |
| Ghana | EPSG:32630 | Sierra Leone | EPSG:32628 |
| The Gambia | EPSG:32628 | Senegal | EPSG:32628 |
| Guinea | EPSG:32629 | Eswatini | EPSG:32736 |
| Guatemala | EPSG:32615 | Chad | EPSG:32633 |
| Honduras | EPSG:32616 | Togo | EPSG:32631 |
| Haiti | EPSG:32618 | Tajikistan | EPSG:32642 |
| India | EPSG:32644 | Timor-Leste | EPSG:32751 |
| Indonesia | EPSG:23845 | Tanzania | EPSG:32736 |
| Jordan | EPSG:32636 | Uganda | EPSG:32636 |
| Kenya | EPSG:21037 | South Africa | EPSG:32735 |
| Cambodia | EPSG:32648 | Zambia | EPSG:32735 |
| Comoros | EPSG:32738 | Zimbabwe | EPSG:32735 |
| Kyrgyz Republic | EPSG:32642 | | |

**Table S9. Model specifications and comparisons based on the Akaike Information Criterion (AIC).**

| Model specification | Degrees of freedom | AIC |
|---|---|---|
| PLAND + $AI_{res}$ | 33 | 231854.4 |
| ns(PLAND,df=2) + ns($AI_{res}$,df=2) | 35 | 231849.9 |
| ns(PLAND,df=2) + $AI_{res}$ | 34 | 231848.6 |
| PLAND + ns($AI_{res}$,df=2) | 34 | 231856.1 |
| ns(PLAND,df=3) + $AI_{res}$ | 35 | 231850.4 |

**Table S10. Comparison of the ranges of key exposure and contextual variables between the urban clusters in 57 LMICs used to estimate the exposure–response relationships and the urban grids in 98 LMICs (in 2015) included in the counterfactual scenario analysis.**

| Variable | 98 countries | | 57 countries | |
|---|---|---|---|---|
| | min | max | min | max |
| PLAND (%) | 0.60 | 100 | 0 | 100 |
| AI (%) | 0 | 100 | 0 | 100 |
| Annual mean $PM_{2.5}$ (μg/m$^3$) | 3.4 | 156.1 | 4.5 | 171.2 |
| Annual mean air temperature (°C) | -10.3 | 34.1 | -13.7 | 31.8 |
| Healthcare accessibility | 0 | 1720 | 0 | 2068 |
| Human Development Index (16) | 0.388 | 0.839 | 0.414 | 0.811 |
| Climate region | tropical, arid, temperate, cold | | tropical, arid, temperate, cold | |

**The 98 countries included in the counterfactual scenario analysis are:** Afghanistan, Algeria, Angola, Bangladesh, Belize, Benin, Bhutan, Bolivia, Botswana, Burkina Faso, Burundi, Cambodia, Cameroon, Cape Verde, Central African Republic, Chad, Colombia, Comoros, Congo, Costa Rica, Cuba, Côte d'Ivoire, Djibouti, Dominican Republic, Democratic Republic of the Congo, Ecuador, Egypt, El Salvador, Equatorial Guinea, Eritrea, Eswatini, Ethiopia, Gabon, Ghana, Guatemala, Guinea, Guinea-Bissau, Guyana, Haiti, Honduras, Indonesia, Iraq, Iran, Jamaica, Jordan, Kenya, Kyrgyz Republic, Lao People's Democratic Republic, Lesotho, Liberia, Libya, Madagascar, Malawi, Mali, Mauritania, Mongolia, Morocco, Mozambique, Myanmar, Namibia, Nepal, Nicaragua, Niger, Nigeria, Pakistan, Palestine, Panama, Papua New Guinea, Paraguay, Peru, Philippines, Venezuela, Rwanda, Senegal, Sierra Leone, Somalia, South Africa, South Sudan, Sri Lanka, Sudan, Suriname, Syrian Arab Republic, São Tomé and Príncipe, Tajikistan, Tanzania, Thailand, The Gambia, Timor-Leste, Togo, Trinidad and Tobago, Tunisia, Turkmenistan, Uganda, Uzbekistan, Viet Nam, Yemen, Zambia, Zimbabwe.

**References**


1. S. Heft-Neal, J. Burney, E. Bendavid, M. Burke, Robust relationship between air quality and infant mortality in Africa. *Nature* **559**, 254–258 (2018).
2. Y. Zou, *et al.*, Fragmentation increased in over half of global forests from 2000 to 2020. *Science* **389**, 1151–1156 (2025).
3. H. Wang, *et al.*, Where and how to invest in greenspace for optimal health benefits: a systematic review of greenspace morphology and human health relationships. *Lancet Planet. Health* **8**, e574–e587 (2024).
4. K. McGarigal, *FRAGSTATS: Spatial Pattern Analysis Program for Quantifying Landscape Structure* (U.S. Department of Agriculture, Forest Service, Pacific Northwest Research Station, 1995).
5. X. Zhang, *et al.*, GLC_FCS30D: the first global 30 m land-cover dynamics monitoring product with a fine classification system for the period from 1985 to 2022 generated using dense-time-series Landsat imagery and the continuous change-detection method. *Earth Syst. Sci. Data* **16**, 1353–1381 (2024).
6. Y.-D. Zhang, *et al.*, Greenspace and burden of infectious illnesses among children in 49 low- and middle-income countries. *Cell Rep. Sustain.* **1**, 100150 (2024).
7. H. E. Beck, *et al.*, High-resolution (1 km) Köppen-Geiger maps for 1901–2099 based on constrained CMIP6 projections. *Sci. Data* **10**, 724 (2023).
8. S. Shen, *et al.*, Enhancing Global Estimation of Fine Particulate Matter Concentrations by Including Geophysical a Priori Information in Deep Learning. *ACS EST Air* **1**, 332–345 (2024).
9. D. N. Karger, P. Brun, F. Zilker, CHELSA-monthly climate data at high resolution. *EnviDat*. https://doi.org/https://www.doi.org/10.16904/envidat.686.
10. Z. S. Venter, A. Hassani, E. Stange, P. Schneider, N. Castell, Reassessing the role of urban green space in air pollution control. *Proc. Natl. Acad. Sci.* **121**, e2306200121 (2024).
11. E. Y. Pfannerstill, *et al.*, Temperature-dependent emissions dominate aerosol and ozone formation in Los Angeles. *Science* **384**, 1324–1329 (2024).
12. D. J. Weiss, *et al.*, Global maps of travel time to healthcare facilities. *Nat. Med.* **26**, 1835–1838 (2020).
13. C. C. Konijnendijk, Evidence-based guidelines for greener, healthier, more resilient neighbourhoods: Introducing the 3–30–300 rule. *J. For. Res.* **34**, 821–830 (2023).
14. R. Burstein, *et al.*, Mapping 123 million neonatal, infant and child deaths between 2000 and 2017. *Nature* **574**, 353–358 (2019).
15. M. Schiavina, *et al.*, GHSL Data Package 2023. *JRC Publ. Repos.* (2023). Available at: https://publications.jrc.ec.europa.eu/repository/handle/JRC133256 [Accessed 23 November 2025].
16. United Nations Development Programme (UNDP). Human Development Index (HDI) [dataset]. Human Development Data Center. Available at: https://hdr.undp.org/data-center/human-development-index#/indicies/HDI. [Accessed April 15, 2026].
